# Transferable Deep Reinforcement Learning With Edge‐Contour‐Depth Fusion for Autonomous Wireless Capsule Endoscopy Navigation

**DOI:** 10.1002/advs.202600008

**Published:** 2026-06-12

**Authors:** Haoxuan Wu, Haitao Gao, Qingyang Liu, Sishen Yuan, Haiyang Fang, Mingwu Su, Baijia Liang, Yongzun Yang, Long Bai, Wenzhen Dong, Dihong Xie, Shijian Su, Jiewen Lai, Shing Shin Cheng, Zhen Li, Xiuli Zuo, Hongliang Ren

**Affiliations:** ^1^ Department of Electronic Engineering The Chinese University of Hong Kong Hong Kong China; ^2^ School of Computer Science and Engineering University of New South Wales Sydney New South Wales Australia; ^3^ Department of Mechanical and Automation Engineering The Chinese University of Hong Kong Hong Kong China; ^4^ Qilu Hospital of Shandong University Jinan Shandong China

**Keywords:** autonomous WCE navigation, deep reinforcement learning, model transferability, sim‐to‐real transfer, wireless capsule endoscopy(WCE)

## Abstract

Wireless capsule endoscopy (WCE) enables painless, minimally invasive visualization of the gastrointestinal tract. Still, its diagnostic potential is limited by incomplete mucosal coverage and poor transferability of existing navigation methods across patient anatomies. We propose a transferable, anatomical landmark‐guided deep reinforcement learning framework for robust autonomous gastric navigation. Leveraging a lightweight edge‐contour‐depth fusion module, our policy operates on stable, low‐dimensional landmark coordinates rather than high‐dimensional video streams. This design effectively bridges the sim‐to‐real visual gap and ensures robustness across diverse anatomies, enabling low‐cost deployment by reducing computational overhead. In simulations across eight patient‐derived models, the method achieves >97% coverage within 50 s, significantly outperforming vanilla Proximal Policy Optimization, Soft Actor‐Critic, and Deep Q‐Network agents by enhancing coverage and minimizing variance. To ensure deployment reliability, a two‐stage sim‐to‐real pipeline supported by an adaptive dynamic programming controller actively mitigates physical disturbances, including actuator latency and peristalsis. Ex vivo experiments across five independent scans demonstrate high coverage stability, achieving a mean coverage of 87% and a 53% reduction in procedure time compared with expert manual control. This study establishes a scalable paradigm for autonomous, high‑coverage endoscopic navigation, advancing the clinical deployment of intelligent WCE systems for GI diagnostics.

## Graphical abstract

This study presents an anatomical landmark‐guided DRL framework for autonomous wireless capsule endoscopy navigation. Using a lightweight edge‐contour‐depth fusion module, it achieves over 97% coverage across diverse gastric anatomies. To ensure reliability, a two‐stage sim‐to‐real pipeline with an adaptive dynamic programming controller mitigates physical disturbances, establishing a promising framework for autonomous gastric navigation.

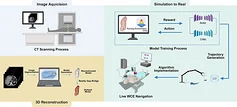

## 1 Introduction

Wireless capsule endoscopy (WCE) has revolutionized gastrointestinal diagnostics by enabling comprehensive, minimally invasive visualization of the entire digestive tract. Unlike conventional approaches, WCE provides unprecedented access to the small intestine and stomach, facilitating the early detection of mucosal lesions and subtle pathological changes that might otherwise be missed [1, 2, 3, 4]. Among these, gastric polyps—sessile or pedunculated protrusions arising from the gastric epithelium or submucosa—are of particular clinical concern due to their malignant potential [5, 6, 7]. The risk of malignant progression is closely tied to histological subtype, underscoring the importance of thorough, systematic mucosal inspection during gastric examination [8, 9]. Maximizing mucosal surface coverage is therefore a central requirement for next‑generation WCE systems, with direct implications for diagnostic reliability and patient outcomes [10].

Achieving complete gastric coverage, however, remains challenging due to the absence of fully autonomous navigation. Current practice relies on manual capsule manipulation within a highly complex and dynamically deforming gastric lumen. Navigation is typically achieved through externally applied magnetic fields, generated via permanent magnets or electromagnetic coils placed around the patient [11, 12, 13]. Early methods, such as hand‑held magnets, offered low precision and were heavily dependent on the operator's skill and judgement [14]. Subsequent technological advances have included robotic‑arm assistance [15, 16, 17], joystick‑controlled electromagnetic platforms offering greater degrees of freedom (DOF) [18], and high‑DOF serial‑link manipulators for real‑time control in fluid‑distended stomachs [19, 20, 21]. While these approaches have improved positioning accuracy and coverage fidelity [22], they still suffer from incomplete automation, restricted DOF, and the inherent complexity of navigating within an unstructured, deformable organ [23]. Consequently, the lack of robust autonomous capabilities continues to limit consistent, operator‑independent gastric coverage.

A further bottleneck is real‑time localization and coverage assessment. Physicians must infer capsule position and coverage status from limited endoscopic imagery, often collected under low illumination and with ambiguous anatomical context [24, 25, 26]. Recent deep learning‑based localization methods that integrate visual, temporal, and motion cues have improved anatomical landmark identification [27, 28, 29], but their performance depends heavily on specific organ geometries, device optics, and dataset diversity, limiting transferability across patient anatomies.

Deep reinforcement learning (DRL) has shown promise for autonomous capsule trajectory tracking and target reaching [30, 31]. However, existing studies adopt a conventional “end‐to‐end” visual DRL paradigm, training policies by feeding high‐dimensional endoscopic images directly into the agent's observation space. Although this approach can achieve high coverage when evaluated within its original training environment [32, 33], this design introduces several critical limitations. First, it causes policies to overfit to the specific morphological and textural features of the training environment, which severely restricts scalability and reduces transferability to unseen geometries. Second, this reliance on raw visual input introduces two further barriers to clinical translation: (1) computational demands for training and deployment, which are infeasible for low‐cost clinical hardware, and (2) vulnerability to the reality gap and visual variations across patients, leading to poor transferability. Since comprehensive patient‐specific training datasets are impractical to obtain, improving transferability by overcoming the limitations of visual‐based DRL is essential for clinical deployment.

**FIGURE 1:**
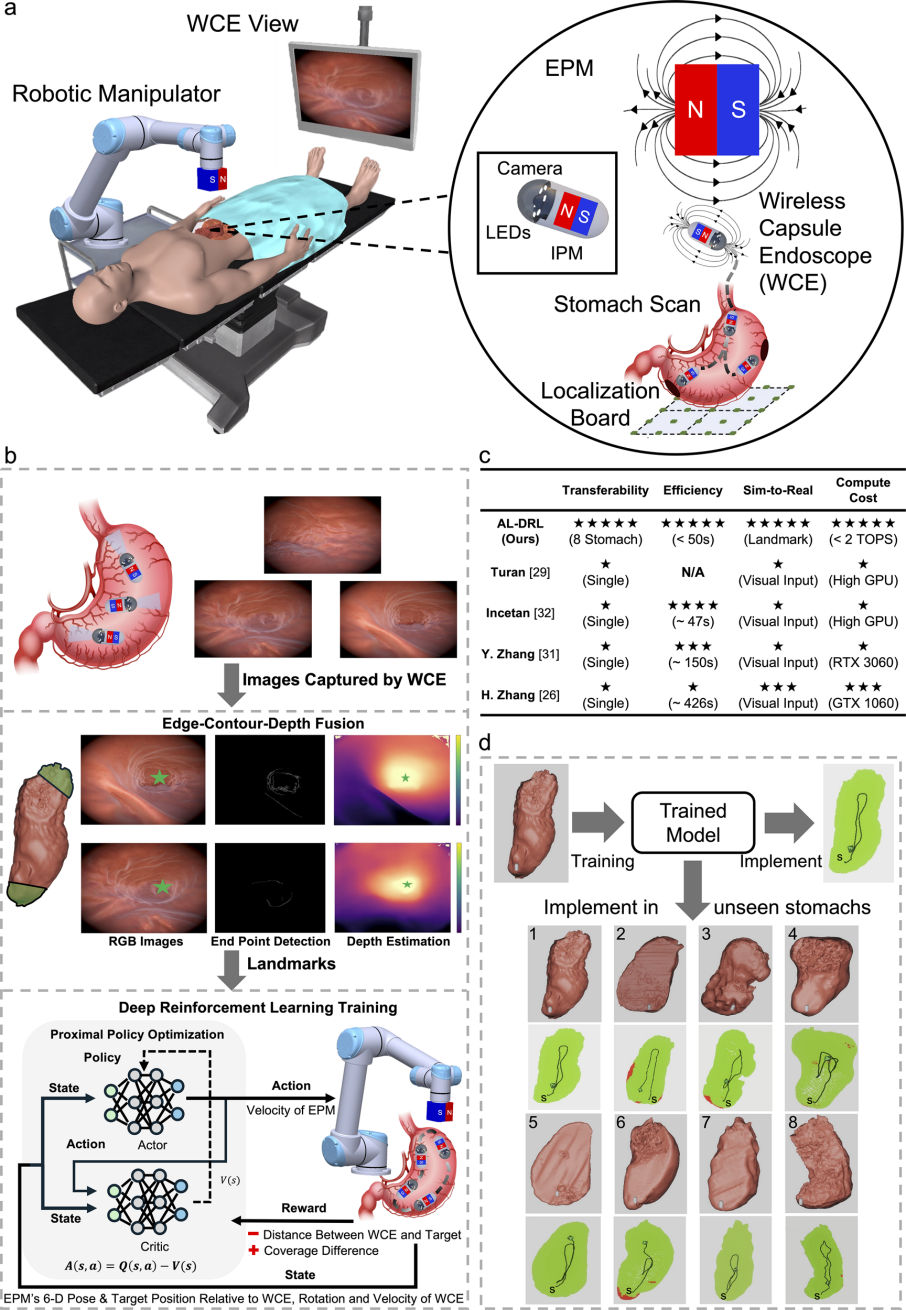
Experimental system and workflow for transferable DRL‐based endoscopic navigation. (a) Schematic of the experimental setup with a single camera capsule inside the stomach and an external 6‐DOF robotic arm actuated via a permanent magnet. (b) AL‐DRL training pipeline comprising image acquisition, preprocessing, and policy learning via PPO. (c) Comparison with State‐of‐the‐Art learning‐based gastric navigation methods. (d) Training and transferability paradigm: DRL agents are trained with landmark guidance on a single stomach phantom and subsequently tested in previously unseen anatomies to assess model transferability; *s* denoted the start point of the trajectory.

Here, we present a unified framework that integrates principled navigation feature selection, state‑of‑the‑art DRL, and robust optimal control to achieve reliable and transferable gastric coverage. Based on the key insight that while mucosal imagery is highly variable, gastric anatomical structures are highly conserved across patients [34]. We therefore establish explicit criteria—universality, distinctiveness, and navigational utility—for selecting anatomical landmarks. This principled approach allows us to replace volatile, high‐dimensional visual data with stable, low‐dimensional landmark coordinates, enabling DRL agents to consistently interpret spatial cues across anatomically diverse stomachs. Building on this foundation, we systematically train and benchmark PPO, SAC, and DQN for autonomous discovery of coverage‑maximizing trajectories in complex gastric environments. Our approach circumvents the challenges of transferability and high computational cost by training a lightweight DRL agent whose policy relies not on raw visual data, but on stable, low‐dimensional spatial coordinates—representing salient visual features and depth cues—extracted via our lightweight edge‐contour‐depth fusion module. This design enhances policy transferability and preserves high performance in anatomically varied and unseen stomach models. To close the sim‑to‑real gap, we further design an optimal control scheme that compensates for actuator latency, sensor noise, and other real‑world disturbances, enabling direct policy deployment on physical WCE platforms with accurate, rapid trajectory execution [35, 36]. Thus, these contributions advance autonomous endoscopic navigation toward clinically viable systems capable of consistent, high‑coverage operation across diverse anatomical and procedural scenarios (Figure 1c).

## 2 Results

### 2.1 Design of Offline AL‐DRL Framework

The offline anatomical landmark‑guided deep reinforcement learning (AL‑DRL) framework adopts a monocular camera‐equipped WCE as the indirectly actuated robotic platform. It integrates a supporting hardware system, a dual‑modal perception module combining visual and six‑degree‑of‑freedom pose information, and a decision‑making module (Figure 1). The hardware system consists of two primary components: an internal section, containing the single‑camera WCE, which captures gastrointestinal images and is equipped with an array of internal permanent magnet (IPM) for magnetic control; and an external section, comprising a 6‑DOF robotic arm (UR5, Universal Robots) with an external permanent magnet (EPM) mounted on the end‑effector, and a magnetic field sensor array. The robotic arm manipulates the EPM to generate magnetic forces and torques that control the capsule's motion, while the magnetic field sensor array provides continuous 6‑DOF pose estimation of the WCE, enabling precise spatial positioning (Figure 1a).

The perception module captures images at 1280 × 720 pixels and 30 frames per second, and obtains 6‑DOF pose estimates of WCE through AMagPoseNet [37], which achieves a translational error of 1.87 ± 1.14 mm, a rotational error of 1.89 ± 0.81°, and a latency of 2.08 ± 0.02 ms. Visual frames and magnetic localization data serve as feedback signals for navigation and control. Within this module, an offline edge‐contour‐depth fusion pipeline integrates three core components—edge detection, contour matching, and a monocular depth estimation neural network—to extract geometric and structural features that enhance environmental perception and support accurate positioning in complex anatomical environments.

The decision‐making module is built on a DRL training architecture in which the navigation policy *π*, parameterized by a neural network, is iteratively refined through agent‐environment interaction to maximize the coverage ratio and minimize the elapsed time. A key feature of this architecture is that the DRL policy *π* does not take high‐dimensional image data as input. Instead, the policy's observation vector is composed exclusively of low‐dimensional state information, including the 3D coordinates of the target landmark (derived from the perception module) and the EPM and capsule's real‐time 6‑DOF poses (Figure 1b). This design drastically reduces the computational load and decouples the control policy from visual domain shifts. At each step, the policy outputs EPM displacement and rotation commands, which directly influence the capsule's trajectory. Training is conducted in a simulated stomach reconstructed from patient CT data to preserve anatomical fidelity (Note S1).

In the simulation environment, the AL‐DRL framework achieves autonomous, high‐coverage navigation across eight distinct patient‐derived stomach models (Figure 1d) through the synergistic integration of the perception, decision, and actuation modules. The process begins with the perception module, which fuses real‐time 6‑DOF pose data with visual priors. These priors are derived from a preprocessing stage that identifies stable, representative gastric landmarks according to established selection criteria—universality, distinctiveness, and navigational utility. These landmarks establish a semi‐deterministic global trajectory, embedding anatomical prior knowledge to structure the complex gastric lumen and guide the agent's exploration. The decision module leverages this prior knowledge to guide policy learning. The agent is trained to systematically traverse the landmarks while locally maximizing mucosal coverage, a behavior explicitly shaped by the reward function:

(1) $$r_{t}=k_{1}\;\left(C_{t}-C_{t-1}\right)-k_{2}D_{t}+R$$

This function incentivizes increases in the coverage ratio *C_t_* via the term *k*_1_(*C_t_* − *C*_*t* − 1_), penalizes Euclidean distance *D_t_* from the current target landmark, and provides a discrete positive reward *R* upon reaching a designated endpoint. Upon reaching a landmark, the agent reorients toward the next, decomposing the complex global coverage task into a sequence of manageable navigation sub‐goals and ensuring systematic inspection of all gastric regions (Figure 4). The actuation module translates the policy's commands into precise magnetic forces and torques via the EPM to control the capsule's motion.

By augmenting the efficiency of point cloud‐based training with pre‐computed landmark and depth features derived from classical image processing and lightweight neural inference, the framework circumvents the need for large scale datasets and computationally intensive training schedules. These embedded visual priors are the key to enhancing transferability, enabling policies trained on a single stomach to be transferred directly to anatomically distinct, unseen stomach models while maintaining high performance.

### 2.2 Transferability Characterization of Vanilla DRL Algorithms in WCE Navigation

**FIGURE 2:**
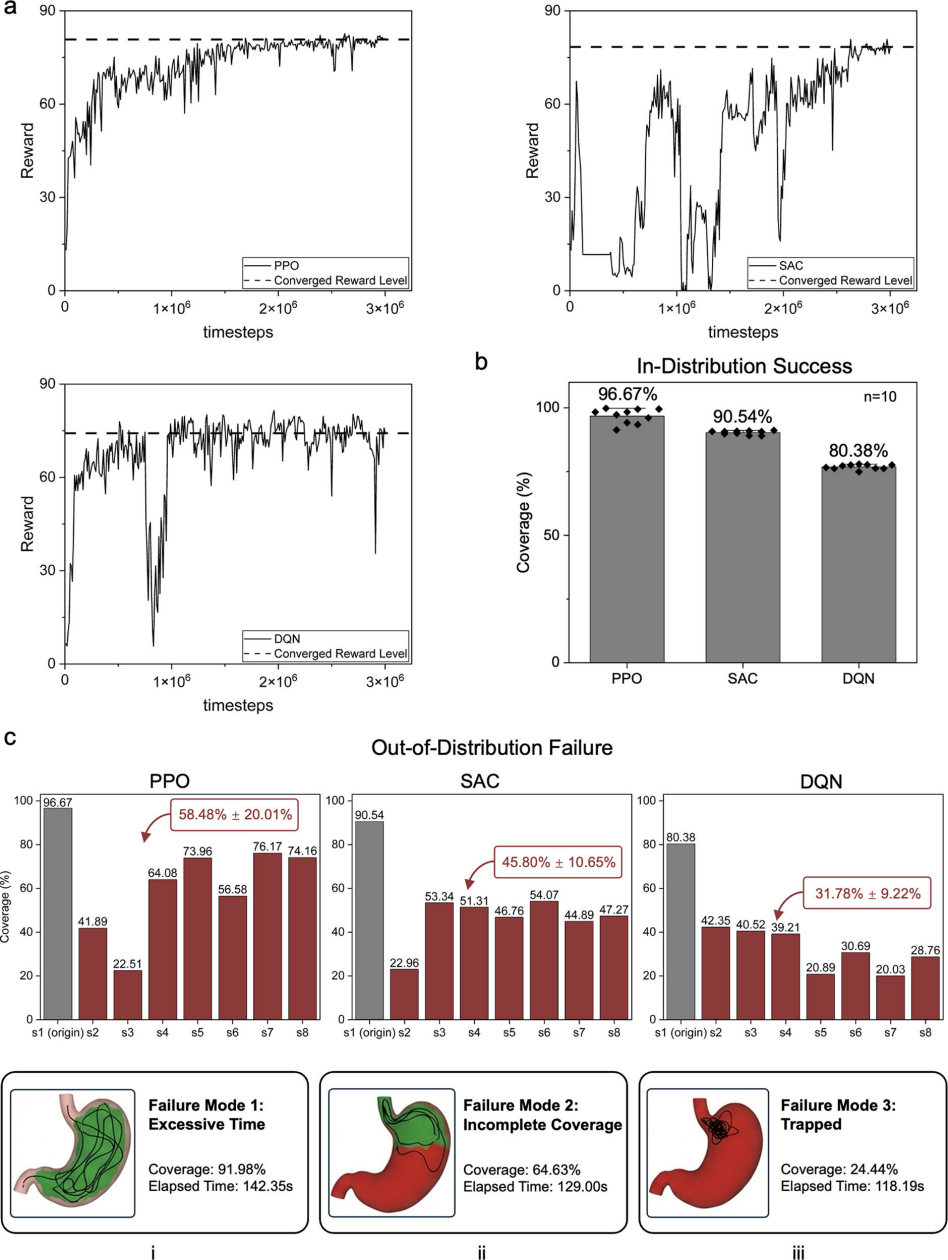
Training performance and transferability of DRL agents. (a) Learning curves of PPO, SAC, and DQN agents during the training phase on a single stomach model. (b) In‐distribution success rates demonstrating that all three algorithms achieve high coverage with small variance across 10 independent trials when deployed in the original training environment. (c) Out‐of‐distribution performance and failure mode analysis in unseen anatomies. The bar charts compare the mean coverage across eight anatomical scenarios (s1–s8), where s1 is the original environment and s2–s8 are unseen structures. The red labels indicate the aggregate performance across the unseen structures (s2–s8), with data presented as mean ± SD. The bottom panels visualize three representative failure modes: (i) Excessive Time, (ii) Incomplete Coverage, and (iii) Trapped in limited regions.

To evaluate the transferability capacity of DRL algorithms for gastrointestinal navigation, we benchmarked three vanilla representative state‐of‐the‐art methods—Proximal Policy Optimization (PPO) [38], Deep Q‐Network (DQN) [39], and Soft Actor‐Critic (SAC) [40]—within an anatomically realistic stomach simulation environment. PPO, widely recognized for its robustness in complex continuous control tasks [41, 42], demonstrated superior stability and learning efficiency during training on a single anatomical model. It achieved a mean reward of 72.52 with a standard deviation (SD) of 10.20, outperforming DQN (mean reward 67.56, SD 14.36) and SAC (mean reward 46.53, SD 25.54) under identical training conditions (Figure 2a). This superiority was further validated in deployment. When the trained policies were executed for ten consecutive trials within the same simulation environment used for training (s1), all three DRL agents demonstrated consistently high mucosal coverage with relatively small standard deviations, indicating that the learned policies were stable and effective within the familiar setting. Specifically, the PPO‐based agent achieved a mean mucosal coverage of 96.67% (SD 2.93%), which substantially outperformed the agents trained with SAC (90.54% coverage, SD 3.75%) and DQN (80.38% coverage, SD 6.01%). These quantitative data confirmed PPO as the most robust and proficient algorithm for single anatomy navigation, establishing it as the baseline for subsequent transferability studies (Figure 2b).

However, this high level of proficiency and consistency proved to be environment‐specific. When the trained PPO policy was applied to previously unseen stomach anatomies (s2–s8), the agent's performance exhibited a dual degradation: a significant reduction in mean mucosal coverage coupled with a dramatic escalation in trial‐to‐trial variability. Specifically, the mean coverage plunged from 96.67% in the training environment to just 58.48% in unseen environments, while the standard deviation surged from a 2.93% to over 20%. This simultaneous collapse in both efficacy and reliability underscores a profound lack of transferability, indicating that the learned policy is highly sensitive to anatomical variations. Similar patterns of performance decay and increased volatility were observed for SAC (45.80% ± 10.65%) and DQN (31.78% ± 9.22%) (Figure 2c; Note S2), further confirming that while these vanilla DRL agents can master a specific environment, they fail to maintain a stable and effective navigation strategy across heterogeneous patient structures.

A detailed analysis of these cross‐anatomy trials revealed distinct and recurring failure modes. For instance, while one agent achieved 91.98% surface coverage, the navigation time increased significantly to 142.35 s (Figure 2c(i)). In other cases, seemingly high area scanning masked poor exploration, with the capsule covering mainly the upper stomach while leaving other regions unobserved, resulting in only 64.63% total coverage (Figure 2c(ii)). Critically, agents could become trapped in localized areas, failing to identify new rewarding trajectories and stagnating at just 24.44% coverage after 118.19 s (Figure 2c(iii)). Our analysis indicates these failures stem from the policy's over‐reliance on the specific geometric features of the training environment; even small changes in the internal spatial distribution of point cloud coordinates were sufficient to impair navigation performance. These limitations reveal a fundamental deficiency in exploratory flexibility, hindering cumulative reward acquisition and navigation efficiency in unseen environments. In a clinical context, the aforementioned transferability failures are untenable, as reliable detection of pathologies—including ulcers, polyps, and tumors—is predicated on comprehensive and evenly distributed mucosal visualization, while efficient navigation is essential for minimizing examination time and patient discomfort [43, 44, 45].

These findings expose a fundamental weakness: policies trained solely on single data modalities, such as 3D point clouds, invariably overfit to the specific anatomical structures of the training environment, leading to a critical lack of adaptability in diverse clinical settings. We therefore posit that achieving robust, transferable autonomous navigation requires moving beyond purely stochastic exploration to a paradigm that integrates prior anatomical knowledge or domain specific biomedical cues—such as geometric or landmark features—into the navigation process. Such structured priors can guide the agent along semi‐deterministic yet adaptable trajectories, enhancing both coverage efficiency and trajectory stability while preserving transferability across previously unseen stomach anatomies [46]. Achieving this requires the use of visual features that are not only distinctive for reliable detection but also ubiquitous enough to serve as consistent navigational references, enabling the capsule to efficiently and systematically explore the gastric environment.

### 2.3 Landmark Involvement and Selection Principle

**FIGURE 3:**
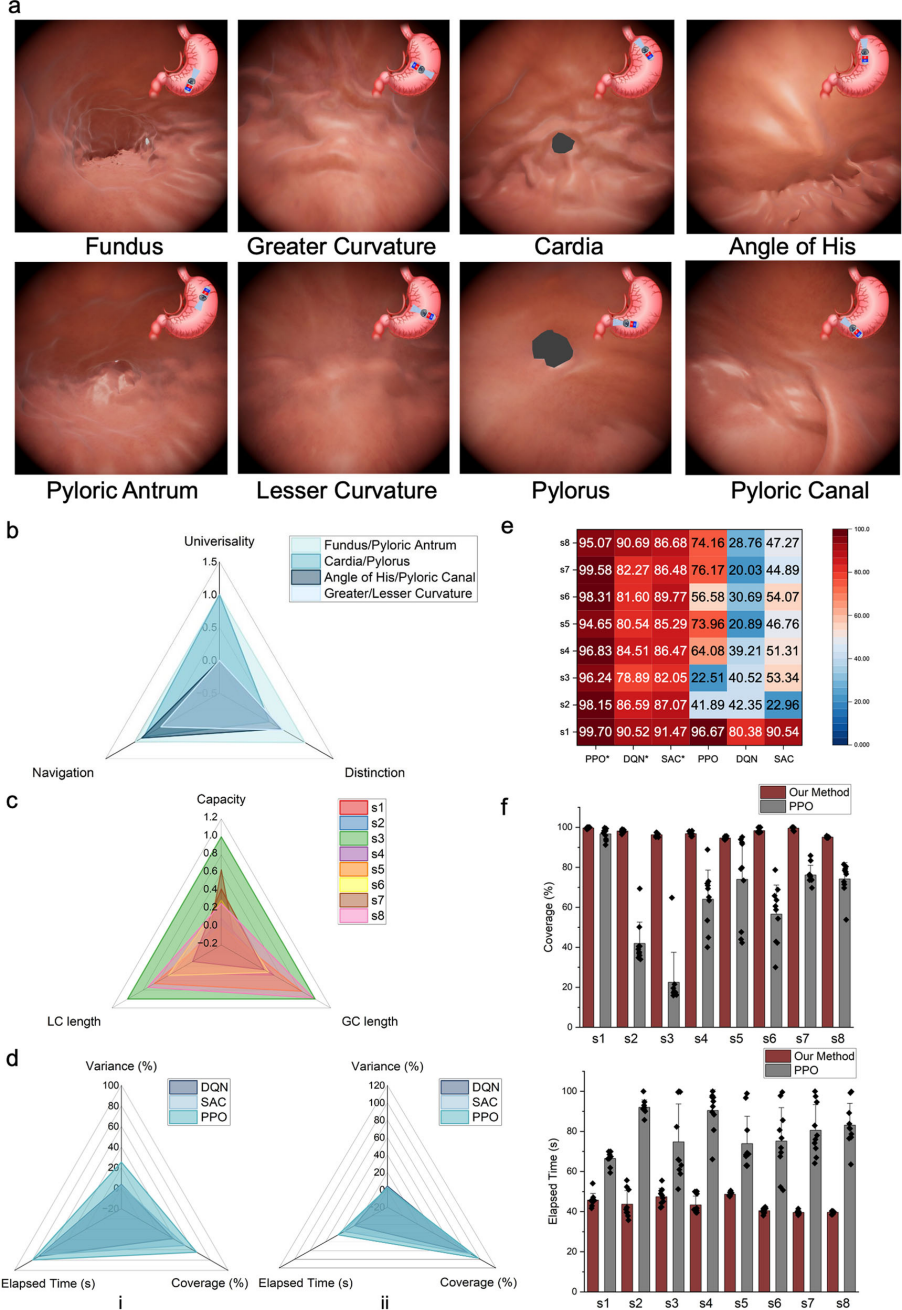
Anatomical landmark selection and transferability characterization of AL‐DRL on WCE navigation. (a) Intra‐gastric views showing candidate landmarks across stomach anatomies. (b) Quantitative evaluation of landmark candidate pairs across three criteria: universality, distinction, and navigation utility. (c) Anatomical diversity of patient‐derived stomach phantoms: eight models reconstructed from CT data, showing variations in capacity, greater curvature (GC) length, and lesser curvature (LC) length. (d) Radar plots comparing key navigation metrics for DRL agents trained without (i) and with (ii) visual landmark guidance across eight distinct stomach models. (e) Heatmap comparing the mean gastric coverage of vanilla versus AL‐DRL‐guided policies across the eight distinct stomach models. (f) Coverage ratio and elapsed time variability across eight stomach models for vanilla PPO versus AL‐DRL.

In gastric environments, most of the mucosal surface lacks consistent or distinctive visual markers, which complicates automated navigation. Nonetheless, certain anatomical landmarks exhibit both visual distinctiveness and structural consistency, making them reliable references for guiding an autonomous capsule through complex intraluminal environments. In this study, four landmark pairs—that is, eight gastric landmarks—were examined (Figure 3a). Features such as the fundus, pyloric antrum, cardia, and pylorus possess pronounced edges and well‐defined contours, enabling consistent detection by image processing algorithms. By contrast, structures such as the greater and lesser curvature, the angle of His, and the pyloric canal often have irregular or poorly defined borders, reducing their reliability when transferring navigation policies between different anatomies. Selecting prominent and dependable landmarks is, therefore, essential to enhance an agent's capacity to transfer across anatomical variability.

We formalize this selection process through three essential criteria: universality, ensuring landmarks are conserved across patient anatomies; distinction, requiring landmarks to be perceptually salient for reliable detection; and navigation utility, demanding that they anchor efficient, coverage‐maximizing trajectories. An evaluation of four candidate landmark pairs across eight patient‐derived stomach models was performed to identify the optimal set based on these criteria.

The criteria of universality and distinction address perceptual reliability. Our analysis revealed that the fundus, pyloric antrum, cardia, and pylorus possess pronounced edges and well‐defined contours, enabling robust identification by image processing algorithms across all models. Quantitative analysis confirmed that the fundus and pyloric antrum, in particular, were detected with 100% accuracy and exhibited maximal edge intensities. In contrast, features such as the greater and lesser curvatures or the angle of His, while anatomically present, lack sharp, consistent borders, rendering them unreliable for policy transfer between anatomies.

Perceptual reliability alone, however, is insufficient. The criterion of navigation utility addresses the functional role of landmarks in guiding exploration. For instance, while the cardia and pylorus are morphologically stable, their spatial locations are suboptimal for anchoring trajectories along the primary gastric axis. Their limited visibility from large portions of the lumen makes them ineffective as global endpoints for systematic inspection. Conversely, the fundus and pyloric antrum are situated at opposing termini of the stomach, providing consistently visible endpoints that define a natural axis for full lumen traversal and efficient path planning.

Based on this multi‐criteria analysis, the fundus and pyloric antrum were identified as the optimal landmark pair, as they uniquely satisfy all three principles of universality, distinction, and navigation utility. This conclusion is quantitatively substantiated by our evaluation, which demonstrated that the fundus and pyloric antrum were detected with 100% accuracy and exhibited maximal edge intensities across all eight patient‐derived stomach models (Figure 3b). Their empirically confirmed perceptual reliability, combined with their strategic anatomical placement for guiding a global traversal of the stomach, solidifies their selection. By embedding this principled landmark selection into the DRL framework, we provide the agent with a robust anatomical map, overcoming a critical limitation of methods reliant on featureless geometric data and establishing a foundation for transferable navigation (Note S3). Accordingly, all subsequent implementations and evaluations of the AL‐DRL framework exclusively utilize the fundus and pyloric antrum as the designated anatomical landmarks.

### 2.4 Transferability Characterization of the Offline AL‐DRL Framework

Having established a principled strategy for landmark selection, the next challenge is to implement a perception module that reliably identifies these landmarks in real time under the severe hardware constraints of a clinical WCE platform. Unlike domains with rich resources, such as autonomous driving, the WCE operates with a low‐power ARM processor (480 MHz), a limited energy budget (< 3 W h), and a strict closed‐loop latency requirement (< 50 ms), rendering computationally intensive deep learning architectures infeasible. To meet these constraints, we developed a computationally efficient edge‐contour‐depth fusion module. This module combines classical, low‐overhead methods—Canny edge detection and Hu moment invariants for rapid landmark identification (10 ms per image pair) (see the “Landmark Identification” section in Methods)—with a pre‐trained, lightweight deep neural network (DispNet, 14.84 M parameters) for accurate monocular depth estimation (RMSE of 0.37 cm) (see the “3D Landmark Localization” section in Methods). This approach proved highly robust, achieving 100% landmark detection reliability across all eight distinct patient‐derived stomach anatomies. This module thus provides the necessary low‐dimensional landmark coordinates for the DRL policy, ensuring the entire navigation framework—from perception to decision‐making—operates efficiently within the WCE's severe computational constraints.

To quantitatively validate our framework, we established a robust and clinically relevant experimental testbed. The evaluation was conducted across eight anatomically diverse gastric models reconstructed from patient CT scans, ensuring the simulation captured realistic variations in gastric size, morphology, and mucosal texture (Table S2 and Note S4). All experiments were performed within a high‐fidelity Unity3D simulator that accurately models the physical interactions between the EPM and the WCE. The simulation environment maintained consistent parameters across all trials, including a monocular camera with a fixed 102° field of view and an initial capsule position 30 cm below the EPM to standardize magnetic coupling. The virtual stomach was positioned so that the capsule initially rested against the gastric wall, replicating gravitational and peristaltic settling observed in vivo. The EPM was manipulated via a UR5 robotic arm using inverse kinematics, with real‐time velocity adjustment to achieve smooth, adaptive capsule control. This comprehensive approach ensured that the evaluation conditions closely mirrored those encountered in practice, providing a reliable basis for assessing robustness and translational applicability.

To assess transferability, seven additional models were introduced, each differing in shape, volume, and curvature parameters (Figure 3c). The simulation environment maintained constant camera settings, robotic arm configurations, and WCE control parameters to ensure comparability across trials. The monocular camera retained a fixed 102° field of view (FOV), and the capsule was initialized 30 cm below the EPM at the start of each run to standardize magnetic coupling and avoid instability from extreme proximity or separation. The virtual stomach was positioned so that the capsule initially rested against the gastric wall, replicating gravitational and peristaltic settling observed in vivo. The EPM was manipulated via a robotic arm using inverse kinematics, with real‐time velocity adjustment to achieve smooth, adaptive capsule control.

Our experimental protocol was designed to rigorously assess transferability. For each of the eight anatomical models, we conducted ten independent trials comparing the performance of standard DRL policies (vanilla PPO, SAC, and DQN) against their counterparts trained using our AL‐DRL framework. The results demonstrate a profound improvement in performance and transferability. When guided by the AL‐DRL framework, the PPO policy's mean coverage across all eight stomachs reached 97.3% with a low SD of 2.0%; this represents a 53.8% increase in coverage and a 45.3% reduction in navigation time compared to its vanilla counterpart (Figure 3f). The benefits extended across all tested algorithms: the SAC policy's coverage increased by 69.1% (to 86.9%) with a 48.9% time reduction, and the DQN policy, previously ineffective for this task, was transformed with a 123.1% increase in coverage (to 84.45%) and a 69.2% time reduction (Figure 3d,e).

**FIGURE 4:**
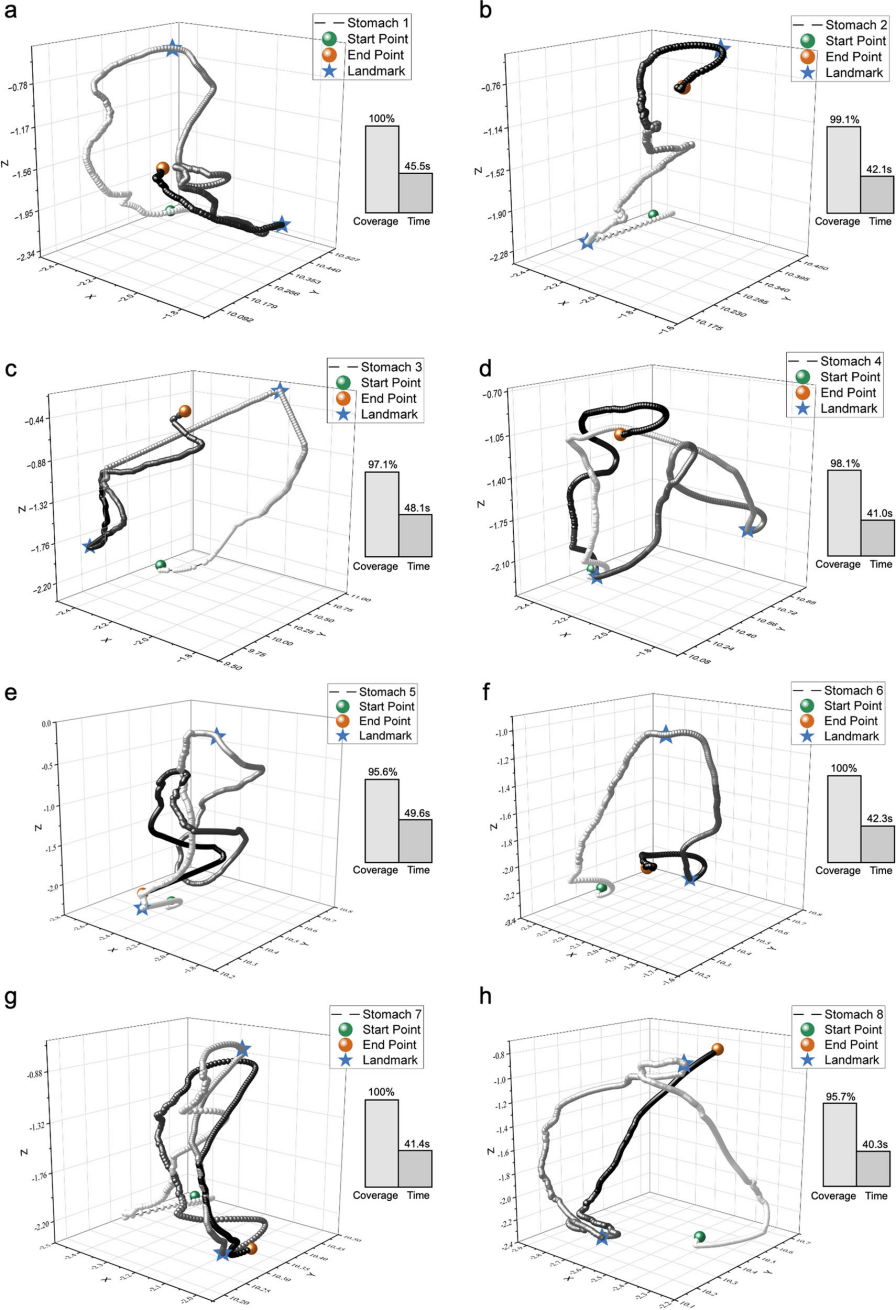
Robust transferability of anatomical landmark‑guided DRL navigation across diverse anatomies. Superimposed trajectories of the WCE in all eight patient‐derived stomach models. Trajectories generated by the AL‐DRL policy with anatomical landmark guidance consistently achieve over 95% gastric coverage within 50 s in each model, underscoring the robustness, efficiency, and reproducibility enabled by visual landmark‐based navigation.

The success of this landmark‐guided approach is visually demonstrated in Figure 4, which shows the 3D navigation trajectories of WCE under the AL‐DRL‐guided PPO policy, serving as a representative result across all eight anatomically distinct models. The figure reveals a consistent, systematic strategy learned by the agent: upon reaching one anatomical landmark, the policy reliably reorients the capsule toward the next. This behavior effectively decomposes the complex global coverage task into a sequence of manageable, landmark‐to‐landmark sub‐goals. By structuring the exploration process, this strategy prevents the agent from becoming trapped in local regions—a common failure mode for vanilla policies—and ensures a systematic inspection of all major gastric regions. The outcome is rapid and comprehensive coverage, with the agent achieving over 95% mucosal inspection in under 50 s across all eight anatomies.

The framework's superior learning efficiency was also evident in training metrics, where the AL‐DRL policy attained a final log‐scaled reward of 8.39, overwhelmingly surpassing the 3.26 achieved by vanilla PPO (Figure S25). These findings, supported by both quantitative performance metrics and direct trajectory visualization, confirm that integrating our lightweight visual perception module with anatomical priors systematically resolves the critical issue of policy overfitting, enabling consistent, rapid, and high coverage autonomous navigation across diverse, clinically realistic environments.

### 2.5 Hybrid Control for Robust Sim‐to‐Real WCE Navigation

Although the AL‑DRL framework performs strongly in simulated gastric environments, direct deployment in real‑world WCE navigation is hindered by sim‑to‑real discrepancies. Our AL‐DRL framework is intrinsically robust to these visual discrepancies, as the navigation policy itself is independent of real‐time mucosal texture and illumination. The primary sim‐to‐real gap we must address is therefore related to hardware dynamics and localization, not visual appearance. These dynamic challenges include idealised zero‑latency actuation in simulation, which contrasts with physical delays of ∼30 ms (motor: 10–20 ms; ROS: 8–10 ms), which increase with payload and mechanical friction. Additional issues include kinematic singularities causing >5 mm positional or >2° orientational errors, and magnetic localisation drift (>7 mm, >5°) from EPM electromagnetic interference. As the DRL model tolerates <5% input noise, such deviations can trigger rapid performance loss and unstable behavior. Therefore, a direct, closed‐loop sim‐to‐real deployment of the DRL policy is infeasible. To address these challenges, we propose a two‑stage control architecture:

1. Patient‑specific digital twin: Reconstructed from CT data to capture gastric geometry and lumen topology. Physical parameters—FOV 102°, focal length 0.57 mm, illumination 1 lux, magnetic moments (EPM: 119.36 Am^2^, IPM: 0.55 Am^2^), reflectivity 0.4, gravity—are empirically calibrated. Realistic collision and friction are modelled in Unity + PhysX using measured Young's modulus and local wall friction. CycleGAN‑based style transfer aligns simulated textures with in vivo imagery.
2. Hybrid execution: Policies are trained in simulation and deployed via offline trajectory planning + online adaptive correction. An optimal path is computed in the digital twin, then followed by a model‑free adaptive dynamic programming (ADP) controller with real‑time visual feedback for deviation correction. Offline planning reduces execution frequency from 200 to 50 Hz, while trajectory smoothing (time/acceleration/jerk minimization) avoids singularities. This confines heavy optimisation to simulation, enabling embedded execution on < 2 TOPS processors.

In the second stage of the framework, motion planning results generated in simulation are transferred to the physical system via an ADP‐based robust tracking controller that compensates for environmental uncertainties and proactively neutralizes hardware latency through forward state prediction (Note S13). Unlike conventional proportional‐integral‐derivative (PID) or fuzzy logic controllers, ADP employs an iterative value‐function approximation to optimize control inputs without requiring explicit system dynamics [47]. This model‑free property makes it particularly suitable for WCE navigation, where complex actuation dynamics and sensor noise limit the applicability of model‑based approaches. The controller minimizes the cost function

(2) $$V=\int_{t}^{\infty }\left(Q\left(x\right)+u^{T}Ru\right)d\tau ,Q\left(x\right)>0,R>0$$

where $x={\left[p_{x}-p_{xd},p_{y}-p_{yd},p_{z}-p_{zd},\phi -\phi_{d},\theta -\theta_{d},\psi -\psi_{d}\right]}^{T}$ represents the position and rotation tracking error state, and *u*  = [*v*, ω]^*T*^  denotes the linear and angular velocity control input of the EPM. Here, *Q*(*x*) penalizes trajectory deviations, while *R* regularizes control effort to ensure smooth actuation.

**FIGURE 5:**
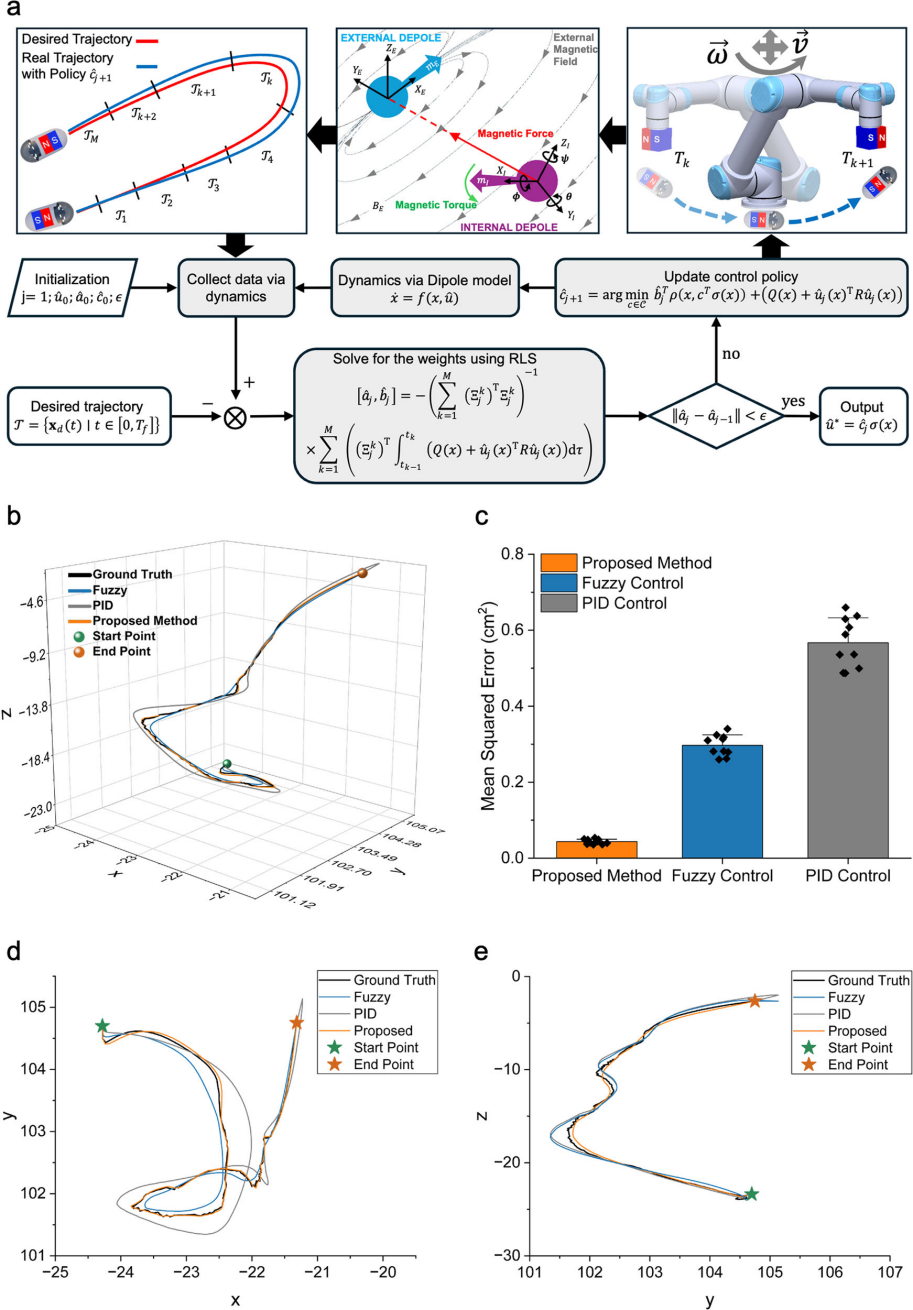
Robust trajectory tracking and control performance under simulated perturbations. (a) Schematic of the offline trajectory planning workflow using a digital twin, coupled with the ADP‐based robust tracking controller for real‐time correction. (b,c) Comparative analysis of mean squared tracking error under simulated perturbations for ADP, PID, and fuzzy control methods. (d,e) Orthogonal projections of the 3D trajectory shown in (b) onto the *x*‐*y* and *y*‐*z* planes.

At each iteration *j*, the control policy ${\hat{u}}_{j}\left(x\right)$ is updated by solving:

(3) $${\hat{c}}_{j+1}=\arg\min_{c\in C}\left\{\underset{\mathrm{Value}\;\mathrm{Gradient}}{\underset{︸}{{\hat{b}}_{j}^{T}\rho \left(x,\;c^{T}\sigma \left(x\right)\right)}}+\underset{\mathrm{Cost}\;\mathrm{Penalty}}{\underset{︸}{\left(Q\left(x\right)+{\hat{u}}_{j}{\left(x\right)}^{T}R\;{\hat{u}}_{j}\left(x\right)\right)}}\right\}$$

where ${\hat{c}}_{j}$ and ${\hat{b}}_{j}$ are neural network weights updated from historical trajectory data via Recursive Least‐Squares (RLS). The term ${\hat{b}}_{j}^{T}\rho \left(\cdot \right)$ estimates the gradient of the value function *V_j_* (*x*), while $Q\left(x\right)+{\hat{u}}_{j}{\left(x\right)}^{T}R\;{\hat{u}}_{j}\left(x\right)$ penalizes tracking errors and excessive control effort. Through successive updates, ADP converges to a policy capable of rejecting disturbances while maintaining stable trajectory tracking (Figure 5a).

Real‑time robustness is further enhanced by coupling ADP with online anatomical landmark detection for dynamic trajectory correction. External disturbances such as magnetic localization drift, gastric peristalsis, and mechanical actuation latency can cause substantial path deviations that threaten task completion. Informed by the reward function's emphasis on endpoint traversal and turning manoeuvres, the system uses real‑time endpoint recognition as a corrective reference. When the deviation from the target exceeds allowable limits, a new path is computed toward the nearest endpoint, and the controller actively steers the WCE back to the intended trajectory. Once the capsule re‑enters a 5 mm proximity zone around the target, the system resumes optimal coverage tracking. This dual‑layer approach mitigates large‑scale deviations and sustains high coverage accuracy despite environmental perturbations.

We validated the ADP framework through comparative experiments against PID (Kp = 0.5, Ki = 0.1, Kd = 0.05) and fuzzy control strategies. An optimal trajectory trained in simulation was executed under each method, while robustness was challenged by introducing magnetic field fluctuations (±2%) and localization noise (positional ±3 mm, orientation ±2°). As shown in Figure 5c, ADP consistently achieved the lowest mean squared tracking error (MSE  =  0.04 cm^2^) compared with PID (0.57 cm^2^) and fuzzy control (0.31 cm^2^), reflecting an 89.5% reduction in error relative to PID. Although a slightly higher error was observed in the *y*‐*z* plane (Figure 5e), overall accuracy and robustness remained superior. In high‑curvature navigation segments (radius < 12 mm), characteristic of abrupt orientation changes required for complete mucosal scanning, ADP maintained a maximum deviation of only 1.3 mm, substantially outperforming PID (5 mm) and fuzzy control (3 mm) (Figure 5b,d).

### 2.6 Real‐World Ex Vivo Validation of the Offline AL‐DRL Framework

**FIGURE 6:**
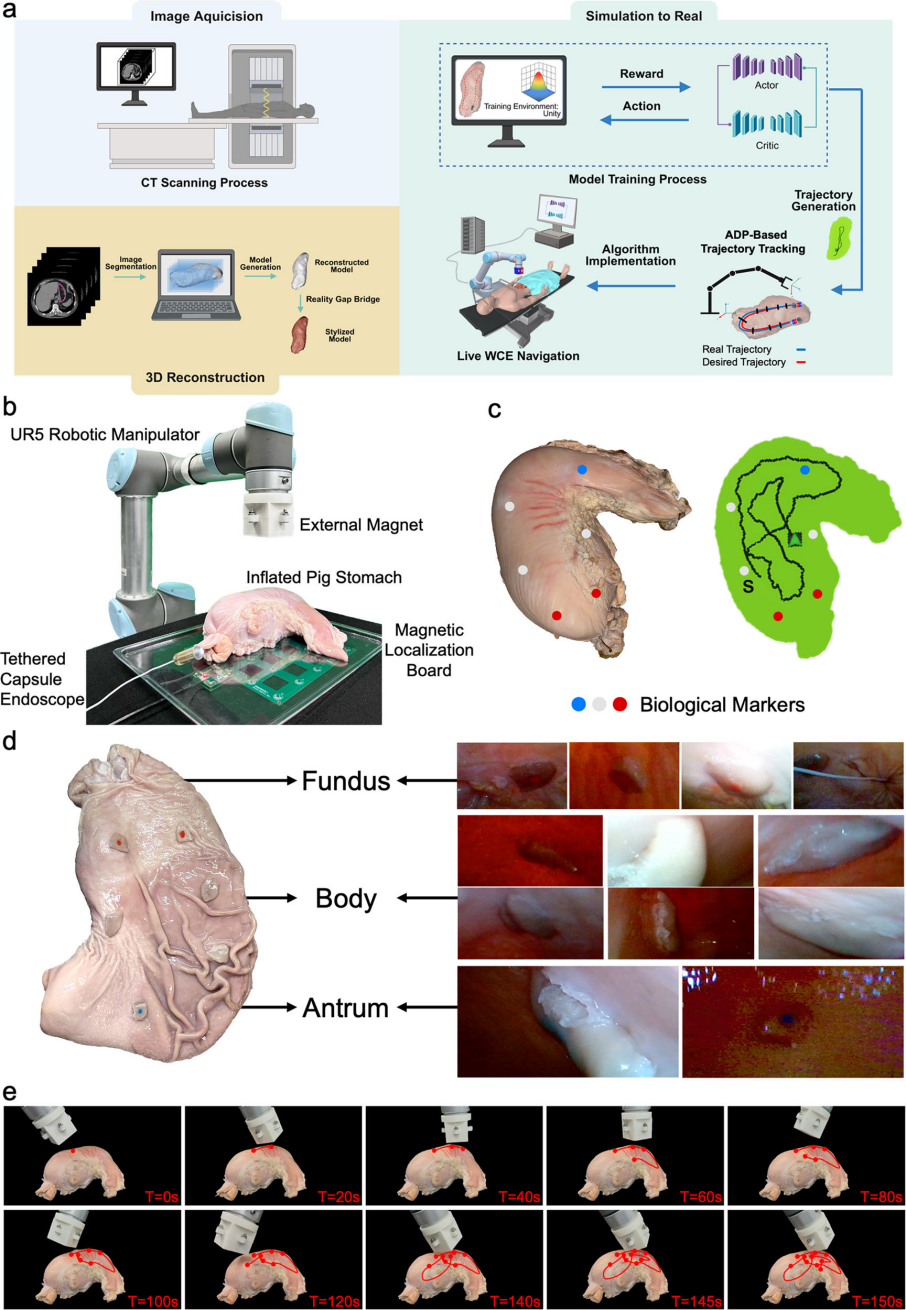
Validation of sim‐to‐real transfer and autonomous navigation within an ex vivo pig stomach model. (a) Simulation‐to‐reality transfer pipeline illustrating the training of a DRL agent in a simulated environment and subsequent deployment in a real‐world WCE procedure. (b) The experimental setup, comprising a UR5 robotic arm that manipulates an EPM to actuate the tethered modular capsule. (c) The ex vivo stomach model, reconstructed from 3D scan data, and the optimal trajectory generated in simulation and subsequently executed on the physical system via the hybrid control algorithm. (d) Representative endoscopic images from the capsule camera, showing markers designating distinct anatomical landmarks during the autonomous scanning process. (e) The trajectory and time stamp of the autonomous scan demonstrate high fidelity to the planned path from the simulation.

To validate the proposed framework under clinically representative conditions, we developed a three‑dimensional multimodal ex vivo test platform (Figure 6b and the “Simulation and Real Environment” section in Methods). The complete data‑to‑execution pipeline is outlined in Figure 6a. Two‑dimensional computed tomography (CT) images of the stomach were first acquired and segmented using advanced algorithms to isolate the organ from surrounding tissues. The segmented slices were reconstructed into high‑fidelity three‑dimensional models using 3D Slicer, and anatomical realism was enhanced by mapping RGB texture information from endoscopic imagery via style‑transfer techniques. The resulting patient‑specific gastric models were then imported into the simulation environment for agent training (Figure 6c), with simulation outputs subsequently translated into real‑world robotic manipulator commands by an adaptive dynamic programming (ADP)‐based controller.

To ensure standardized performance assessment, 12 biological markers, crafted from excised gastric tissue to simulate tumors, were strategically distributed throughout the ex vivo pig stomach. To ensure a comprehensive and uniform assessment, the markers were placed on the surfaces of the three primary gastric regions—the fundus, body, and antrum—to provide fixed references for quantitative coverage and trajectory accuracy. Specifically, four markers painted red were placed in the fundus, six unpainted markers were placed in the body, and two markers painted blue were placed in the antrum. Figure 6d depicts the markers layout on one side; an identical, symmetrical arrangement was placed on the opposing side. The 3D stomach model reconstructed from a scanner is imported into Unity as the training environment. To establish a high‐fidelity digital twin of the experimental setup, our simulation platform was developed in the Unity engine. The physical parameters of every component—including dimensions, mass, magnetic properties, and camera intrinsics—were meticulously configured to match their real‐world values, thereby minimizing the sim‐to‐real gap. After AL‑DRL training, the optimized trajectory was deployed in reality with the UR5 arm. To achieve the target linear and angular velocities for the EPM, the corresponding joint velocities of the manipulator were calculated via inverse kinematics using the manipulator Jacobian (Note S5). During this process, a manipulability index was maintained above 0.1 to avoid singularities. Figure 6d shows the anatomical distribution of the markers and the corresponding WCE images acquired during navigation, confirming successful detection in all gastric regions.

The scanning protocol (Figure 6e) prescribed a serpentine trajectory designed for comprehensive surface coverage. The path originated in the gastric body, followed the greater curvature to the antrum and pylorus, returned along the lesser curvature toward the fundus, and finally swept back toward the gastric body to complete the inspection. Orientation adjustments maximized mucosal exposure throughout. The first segment along the greater curvature took 40 s, covering about one‑third of the gastric surface; the second, from the antrum along the lesser curvature to the mid‑body, lasted 80 s due to a longer distance and a required 180° turn; the final segment, covering the fundus and remaining body, took 40 s with a similar rotation event despite a shorter path.

Reliability and repeatability tests over five independent scans yielded an average coverage ratio of 87% (number of captured markers: 12/12, 10/12, 11/12, 9/12, 11/12) with a completion time of 139.8 ± 10 s (coefficient of variation 6.5%). In comparison, a trained human operator using a joystick required 298.4 ± 30 s (coefficient of variation 7.1%) for equivalent coverage, representing a 53% time reduction with the autonomous system. This improvement stems from the ADP controller's stability, particularly in regions of high curvature (radius < 20 mm), where tracking accuracy remained at 1.5 mm. The singularity‑avoidance constraint prevented abrupt robotic arm movements, ensuring smooth actuation. Collectively, these results demonstrate the potential of the proposed platform as a promising framework for autonomous gastrointestinal navigation. The consistent performance across diverse simulations and initial physical tests suggests a viable path toward reducing operator dependency through intelligent endoscopic robotics.

## 3 Discussion

Here, we present an autonomous WCE area‑coverage framework that integrates DRL, anatomical landmark detection, and monocular depth estimation into a cohesive control system. Conventional RL‑based navigation approaches often show limited transferability across diverse, patient‑specific stomach anatomies, making retraining for each case both computationally demanding and economically impractical in clinical workflows. To address this constraint, we selected PPO—which demonstrated strong single‑model navigation performance—and enhanced its transferability by streamlining the training pipeline and embedding visual feature extraction mechanisms that produce stable, anatomically informative representations. The resulting framework consistently achieved more than 97% mucosal coverage within 50 s across eight anatomically distinct stomach models, effectively eliminating the coverage penalty typically observed when transferring RL agents between heterogeneous anatomies. This performance surpasses point‑based and geometry‑only approaches, establishing a new benchmark for autonomous endoscopic navigation.

Central to this capability is our framework's core architectural design, which deliberately circumvents the computationally demanding and inherently brittle “end‐to‐end” paradigm common in visual‐DRL systems. This conventional approach, which feeds high‐dimensional, real‐time endoscopic imagery directly into the policy, suffers from two critical translational barriers: (1) it demands substantial computational resources (e.g., high‐performance GPUs) that are infeasible for low‐cost, real‐time clinical deployment, and (2) it is fundamentally vulnerable to domain shifts, failing to transfer when faced with the vast differences in mucosal appearance across patients. Our framework, in stark contrast, severs this dependency. The policy is trained to operate exclusively on low‐dimensional 3D coordinates derived from pre‐identified anatomical landmarks. Guided by our principled landmark selection criteria (universality, distinction, and navigational utility), this design is the key to overcoming both aforementioned barriers, yielding profound advantages for clinical translation. First, it renders the navigation task computationally tractable, enabling efficient training and, more importantly, high‐speed deployment on low‐cost, power‐constrained hardware. By optimizing the execution frequency at 50 Hz, we balance efficiency with stability: higher rates are redundant given the 30 ms physical latency, whereas lower rates cannot sufficiently reject real‐time disturbances, such as magnetic drift or peristalsis, leading to tracking failures in high‐curvature segments. This ensures that corrective actions maintain the required control accuracy for robust navigation while minimizing the computational burden on low‐power hardware. Second, and most critically, it achieves robust transferability by making the policy intrinsically invariant to the visual domain shifts that plague end‐to‐end methods. The policy's performance becomes independent of the vast differences in mucosal appearance across patients, ensuring consistent operation across diverse patient anatomies. By decoupling the complex perception challenge from the real‐time control policy, our framework achieves superior robustness and scalability. This anchoring to stable anatomical coordinates, rather than transient visual features, drastically reduces the dimensionality of the policy's observation space, simplifying optimization and ensuring predictable, transferable behavior.

There are, however, notable limitations. The current endpoint‑matching algorithm is sensitive to variations in illumination and affine transformations, leading to occasional detection errors when landmarks are imaged from extreme viewpoints or under challenging lighting. While additional reliability tests demonstrate that our perception module consistently identifies landmarks under off‐axis tilts and dim lighting, we acknowledge that extreme in vivo artifacts (dense mucus coverage or intense specular reflections) could still degrade real‐time detection. To address this, we will integrate a proactive enhancement layer EndoUIC into the recognition module to mitigate detection failures caused by intense reflections or uneven lighting, thereby enhancing overall robustness [48]. To ensure safety during perception failures, the system employs temporal prediction for transient occlusions via ADP controller (<500 ms) and an active search mode via in−place rotation for sustained losses (>2 s), with a manual fallback alert as a final safeguard. More resilient computer vision methods that maintain accuracy across a broader range of intraoperative conditions are needed to address this weakness.

Furthermore, it is important to acknowledge the translational gaps introduced by the current tethered physical testbed compared to a fully wireless clinical scenario. From a dynamics perspective, while our ex vivo experiments utilize a highly flexible tether with negligible macroscopic actuation resistance (∼0.04 N mm), the tether intrinsically acts as a stabilizing “tailfin.” It provides mechanical damping that suppresses high‐frequency oscillations, stick‐slip friction‐induced chatter, and micro‐tumbling, masking the highly non‐linear posture dynamics and complex 6‐DOF tumbling that a free capsule would exhibit. From an imaging perspective, the tethered setup enables 30 fps image acquisition, whereas practical battery‐powered clinical capsules typically operate at lower frame rates (e.g., 2–3 fps) to conserve energy. Operating at such low frame rates would not significantly degrade navigation accuracy, as our ADP‐based controller primarily tracks offline‐learned trajectories and uses real‐time vision only for periodic correction. However, it would inevitably increase the total scanning time. To avoid incomplete anatomical scanning and prevent sweeping past landmarks between sparse visual frames during active recoveries, the EPM actuation speed should be correspondingly reduced. Future improvements will rely on micro‐hardware advancements to enable higher‐frame‐rate transmission within clinical power constraints. Additionally, regarding magnetic localization, while the system employs active compensation for EPM interference, the reliance on an analytical dipole model presents inherent accuracy limitations when encountering non‐linear environmental distortions (e.g., hard‐iron/soft‐iron effects) or dynamic noise from the 6‐DOF robotic arm.

In addition, although the system demonstrates strong transferability across healthy anatomical variants, validation in larger patient cohorts—especially in cases with pathological alterations or atypical morphology—is essential to confirm clinical robustness. Under extreme pathological conditions like large hiatal hernias, where landmark spatial relationships may be significantly altered, the framework ensures safety through a confidence‐based detection mechanism: if the matching similarity score (*D*) falls below a reliable threshold, the system triggers an anomaly alert to prevent erroneous navigation. Furthermore, the low‐level ADP controller's mechanical constraints and the possibility of immediate manual intervention provide redundant safety layers to handle such severe morphological distortions. For the fundamental DRL algorithm, the implemented architecture is based on recurrent neural networks (RNN), which is ineffective for long‐term dependencies and only indirectly accesses the past via a compressed hidden state.

Future work will focus on enhancing the policy's long‐term reasoning capabilities and its adaptability to more dynamic clinical scenarios, while preserving the framework's core computational advantages. Replacing RNN with a Transformer‐based architecture—which excels at modelling complex sequences—could significantly improve the agent's ability to optimize long‐horizon trajectories. Critically, such a model would still operate on the low‐dimensional sequence of coordinate and pose data, maintaining the low‐cost, transferable nature of our approach. We also plan to address the transition to a wireless state as a critical translational boundary. This involves updating our training pipeline with stochastic noise models to simulate wireless instability and enhancing the ADP controller to specifically address high‑frequency perturbations and the reduced damping inherent to tether‑free boundary conditions. To further mitigate unpredictable environmental magnetic noise, we intend to integrate an anti‑interference framework based on MagRobustNet [49]. By utilizing self‑supervised anomaly detection and measurement recovery, this framework has demonstrated a 76.2% improvement in position accuracy, ensuring reliable tracking in complex clinical settings.

Additionally, we plan to extend the framework from pre‐defined landmark traversal to dynamic, target‐reaching capabilities, enabling the agent to navigate to specific 3D coordinates of a pathology (e.g., a polyp) identified intraoperatively. To enhance clinical universality, we will expand the training pipeline by incorporating a broader range of pathological gastric models featuring severe morphological distortions, such as hiatal hernias or massive intraluminal compressions. This expansion will enable the AL‐DRL policy to adapt to atypical anatomical priors where standard spatial relationships are disrupted. Future efforts will develop more robust, adaptive target identification methods and incorporate recovery strategies for navigating in atypical or pathological anatomies where targets may be obscured.

## 4 Methods

### 4.1 Simulation and Real Environment

The simulation environment was developed in Unity (version 2022.3.32f1) as a real‐time 3D platform, using the built‐in NVIDIA PhysX engine to model rigid‐body dynamics with both discrete and continuous collision detection. DRL training was supported through integration of the ML‐Agents toolkit (release 20). A 6‐DOF Universal Robots manipulator served as the simulated actuator mounting an EPM to control the WCE. Eight stomach models derived from patients were imported into Unity as primary test environments. As these virtual stomach models are represented by point clouds, the coverage ratio (*C_t_*)—a key metric for training—is defined as the percentage of the total point cloud points scanned by the capsule's camera. To ensure clinical relevance, a point is only recorded as “scanned” if its distance from the camera lens is within a 30 mm threshold, which is informed by clinical evidence demonstrating that magnetically controlled capsule endoscopy (MCE) achieves an overall diagnostic accuracy of 93.4% within a 0–60 mm viewing range [50]. Physical parameters were meticulously calibrated to mirror real‐world conditions: gravitational acceleration was set to 9.81 m/s^2^, the EPM magnetic moment to 119.36 Am^2^, and the IPM magnetic moment to 0.55 Am^2^. Friction coefficients were set to 0.1 for the gastric wall and 0.5 for the capsule surface. Optical properties were configured with a monocular camera field of view of 102°, a focal length of 0.57 mm, material reflectivity of 0.4, and ambient illumination of 1 lux. To simulate gastric peristalsis, wall friction coefficients were regionally varied and subjected to random perturbations during runtime (Note S6).

The physical testbed (Figure 6b) comprised a 6‐DOF Universal Robots arm manipulating an EPM via a custom‐designed 3D‐printed container that mounted a cubic NdFeB magnet (50 × 50 mm) at the end‐effector, providing an operational workspace radius of 850 mm. The custom WCE measured 12 mm in diameter and 35 mm in length, integrating an OV9734 fibre‐optic imaging module (1280 × 720 pixels at 30 fps, 102° FOV) and an IPM formed by four orthogonal NdFeB pole groups. The capsule was enclosed in a 3D‐printed clear resin sheath (Young's modulus 4 GPa, friction coefficient 0.5, tensile strength 70 MPa). This modular design, costing under $50 to assemble, replaced commercial WCE units that cost over $1000 and lack real‐time video. A low‐modulus tether (2 mm diameter, Young's modulus 10 MPa) preserved realistic capsule kinematics, with dynamic torsion tests showing an added torque of only 0.04 N·mm under ±180° rotation—around 10% of the 0.35 N·mm torque generated by the EPM. Further experimental characterization across a 25–125 mm range confirmed that magnetic torque remains consistently above the tether's resistive threshold, reaching the standardized working value of 0.35 N mm at approximately 90 mm (Note S7).

The scanning medium was an ex vivo pig stomach measuring 31.2 cm in length and 14 cm in maximum width. An air pump was used to inflate the stomach to a distended state to ensure a realistic scanning environment. Twelve biological markers, crafted from excised gastric tissue to simulate tumors, were affixed to the internal ex vivo pig stomach surface—four designated by red marks in the fundus, six unmarked markers in the body, and two designated by blue marks in the antrum. Capsule localization was achieved with a 5 × 5 magnetometer array (LIS3MDL, STMicroelectronics; ±1200 µT range) mounted on a custom localization board with 40 mm sensor spacing. This array resolves the capsule's 6‑DOF pose using AMagPoseNet (Note S8). Data acquisition was handled by an STM32H743 microcontroller, with real‐time transmission to a host PC. To compensate for magnetic interference from the EPM, its real‐time pose—calculated via robotic forward kinematics—is used in a magnetic dipole model to compute its interference field, which is then subtracted from the sensor array's total measurements to isolate the pure signal from the capsule's internal magnet (Note S9).

### 4.2 Landmark Identification

To enable efficient landmark identification under constrained computational resources and in the context of point cloud‐based stomach modelling, the image processing module is designed to identify and match anatomical landmarks using a template‑driven approach. The module comprises two main stages: edge detection and feature matching.

For edge detection, we implement the Canny method, a gradient‑based algorithm for delineating object boundaries. Given a grayscale input image *I*(*x*,  *y*), horizontal and vertical gradients are computed via Sobel filters as

(4) $$G_{x}=\frac{\partial I}{\partial x}\;,\;\;G_{y}=\frac{\partial I}{\partial y}\;$$

where *G_x_* and *G_y_* are the gradients along the *x* and *y* axes respectively. The gradient magnitude and direction are obtained as:

(5) $$G=\sqrt{G_{x}^{2}+G_{y}^{2}}\;,\;\theta =\arctan\left(\frac{G_{y}}{G_{x}}\right)\;$$

To suppress high‑frequency noise, we apply a Gaussian blur with a (9 × 9) kernel before edge detection. The Canny operator is then applied with lower and upper thresholds set to 10 and 20, respectively.

Following edge extraction, we adopt a contour‑based shape matching method to assess similarity between detected endpoints in the target (or distractor) image and a pre‑defined template. From each binary edge map, external contours are identified by locating the outermost connected boundary points. Each contour is simplified to preserve essential geometric properties while reducing data redundancy. Let $C_{1}$ and $C_{2}$ denote the contour sets from images *I*_1_ and *I*_2_, respectively. To enhance robustness against noise, only the most prominent contour—defined as the one enclosing the largest area—is selected from each set:

(6) $$C_{1}^{*}=\arg\max_{C\in C_{1}}\mathrm{Area}\;\left(C\right),\;\;C_{2}^{*}=\arg\max_{C\in C_{2}}\mathrm{Area}\;\left(C^{\prime }\right)$$

Shape similarity is quantified using a moment‐based descriptor derived from the seven Hu moments $H_{1},H_{2},\text{…},H_{7}$, which are invariant to translation, rotation, and scaling. The similarity score between the selected contours is defined as

(7) $$D\;\left(C_{1}^{*},C_{2}^{*}\right)=\sum_{i=1}^{7}\left|\frac{1}{\log H_{i}^{\left(1\right)}}-\frac{1}{\log H_{i}^{\left(2\right)}}\right|$$

where $H_{i}^{\left(1\right)}$ and $H_{i}^{\left(2\right)}$ are the *i*‐th Hu moments computed from $C_{1}^{*}$ and $C_{2}^{*}$, respectively. A lower value of *D* indicates greater shape similarity.

Comparative evaluations with alternative edge‑detection and contour‑matching strategies (Note S10) confirmed that the proposed combination yields the most reliable and computationally lightweight performance, meeting the operational constraints of real‑time WCE navigation. To ensure practical reliability in diverse clinical settings, we further evaluated the module's performance under non‐ideal conditions, including moderate off‐axis tilts and dim‐light environments. The module demonstrates consistent landmark identification and depth calculation accuracy even when features are positioned far from the optical center (Note S12).

### 4.3 3D Landmark Localization

To determine the precise three‑dimensional coordinates of a navigation endpoint, we employed a deep neural network‐based monocular depth estimation model, DispNet, configured within the Endo‑SfMLearner framework [51]. The model receives a single monocular RGB frame *I_i_* captured by the capsule camera and outputs a disparity map *D_i_*, effectively augmenting the original image with per‑pixel depth information. The encoder‐decoder architecture of DispNet (Note S11) was selected for its ability to produce dense and smooth disparity maps under the low‑texture, specular, and variable‑illumination conditions typical of endoscopic imaging. The network was trained using a loss function that integrates geometry consistency, brightness‑aware photometric matching, and smoothness constraints, jointly optimised to improve both the accuracy and robustness of estimated disparities.

Geometry Consistency Loss (*L_GC_*) enforces agreement between forward‑warped disparities $D_{i}^{w}$ and corresponding backward disparities *D*_*i* + 1_, penalizing mismatches via the relative difference metric

(8) $$D_{\mathrm{diff}}\;\left(p\right)=\frac{\left|D_{i}^{w}\left(p\right)-D_{i+1}\left(p\right)\right|}{D_{i}^{w}\left(p\right)+D_{i+1}\left(p\right)}\;$$

where *p* indexes pixels and $D_{i}^{w}\left(p\right)$ denotes the disparity at *p* in frame *i* forward warped into frame *i* + 1. The loss is averaged over all pixels in the set *P*:

(9) $$L_{GC}=\frac{1}{\left|P\right|}\;\sum_{p\in P}D_{\mathrm{diff}}\left(p\right)$$

Brightness‐Aware Photometric Loss (*L_p_*) measures the residual between the reference image *I_i_* and a synthesized view (${\hat{I}}_{i}$) after applying an affine brightness correction *T_b_* (·), thereby compensating for lighting variation. This term blends an $\ell_{1}$ photometric error with a structural similarity index (SSIM) term for enhanced perceptual robustness:

(10) $$L_{p}=\frac{1}{\left|P\right|}\;\sum_{p\in P}\left({\left|\left|T_{b}\left({\hat{I}}_{i}\right)\left(p\right)-I_{i}\left(p\right)\right|\right|}_{1}+\lambda_{1}\frac{1-\mathrm{SSIM}\left(T_{b}\left({\hat{I}}_{i}\right),I_{i}\right)}{2}\right)$$

where

(11) $$T_{b}\;\left({\hat{I}}_{i}\right)=a_{i}\;{\hat{I}}_{i}+c_{i}w,\;M=1-D_{\mathrm{diff}}$$

and λ_1_ controls the SSIM contribution.

Smoothness Loss (*L_s_*) regularises disparity values to promote spatial coherence while preserving depth discontinuities along image edges. By weighting the disparity gradient ∇*D*(*p*) by an exponential function of the image gradient magnitude, the loss emphasises smoothness in low‑texture regions:

(12) $$L_{s}=\sum_{p\in P}{\left(e^{-\left|\nabla I\left(p\right)\right|}\cdot \left|\nabla D\left(p\right)\right|\right)}^{2}\;$$

The total loss is a weighted sum of these three terms:

(13) $$L=\alpha_{p}\;L_{p}+\beta_{s}L_{s}+\gamma_{GC}L_{GC}$$

where α_*p*_, β_*s*_, and γ_*GC*_ balance the contributions of photometric, smoothness, and geometric consistency constraints.

This composite formulation ensures that depth estimates are accurate, geometrically consistent, and resilient to lighting variability. In our implementation, we employed a pre‑trained DispNet model, achieving a root mean square error (RMSE) of 0.37 cm on a desktop platform equipped with a 32 GB AMD Ryzen 5 5600 six‑core processor and an NVIDIA GeForce RTX 4060 GPU, meeting real‑time inference requirements for intraoperative navigation.

### 4.4 EndoSLAM Dataset

DispNet was pre‑trained using data from the EndoSLAM dataset [51], a large‑scale monocular depth estimation benchmark comprising 42 700 frames. The dataset integrates ex‑vivo porcine gastrointestinal organ recordings with synthetic capsule endoscopy sequences generated in the Unity simulation environment, covering anatomical regions including the colon, stomach, and small intestine. Each sequence includes pose‑aligned depth maps, obtained via a combination of computed tomography (CT) imaging and high‑accuracy 3D scanning, alongside corresponding video streams captured by both capsule endoscopy and conventional endoscopic cameras.

Although labelled and calibrated, EndoSLAM is designed primarily for unsupervised monocular depth estimation, where models learn depth indirectly from photometric consistency between consecutive frames rather than from direct pixel‑wise supervision. This structure facilitates domain adaptation, as the dataset supports the joint evaluation of 3D reconstruction and simultaneous localisation and mapping (SLAM) techniques in both synthetic and physical environments. Its composition is particularly suited to simulation‑to‑real transfer learning, enabling pre‑trained models to transfer effectively to real‑world endoscopic imagery for robust geometry prediction.

### 4.5 Training Approach and Workflow

For AL‐DRL training, we employed the PPO algorithm implemented within the Unity ML‑Agents framework. ML‑Agents integrates natively with the Unity 3D simulation engine, supporting both headless (terminal‑based) training and interactive visual rendering, thereby enabling flexible control over computational resources and debugging. The hyperparameters were tuned (Table 1) to improve capsule performance within a given stomach model. For landmark‑guided training, the two anatomical endpoints were first localised using the Image Processing Module. The agent was then trained using a reward function specifically formulated for the offline AL‑DRL framework, allowing precise evaluation of landmark‑guided WCE navigation performance in the anatomical model. In this setup, the observation vector—which serves as the direct input to the DRL agent's policy network—is composed of the 3D coordinates of the target landmarks, combined with real‐time spatial and temporal information from WCE and EPM. This custom reward function, defined via C# script within the Unity environment, then uses this real‐time information to calculate the DRL reward for training (Algorithm 1).

**TABLE 1:** Hyperparameters in the PPO, SAC, and DQN Algorithms.

Parameter	PPO	SAC	DQN
Trainer	PPO	SAC	DQN
Batch Size	1024	1024	64
Buffer Size	4096	512000	50000
Hidden Units	128	128	128
Learning Rate	0.001	0.0005	0.0003
Learning Rate Schedule	Linear	Linear	constant
Max Steps	3000000	3000000	3000000
Memory Size	512	512	10000
Num Layers	2	2	2
Time Horizon	1024	1024	1024
Sequence Length	64	64	N/A
Summary Freq	10000	10000	1000
Gamma	0.99	0.99	0.99
Num Epoch	5	N/A	N/A
Lambda	0.95	N/A	N/A
Epsilon	0.2	N/A	1.0
Beta	0.005	N/A	N/A
Tau	N/A	0.005	0.005
Exploration Schedule	N/A	N/A	Linear
Exploration Initial Eps	N/A	N/A	0.8
Exploration Final Eps	N/A	N/A	0.05

Algorithm 1 **Agent training in each episode**

**Table:** 

**Require**:	
Position of the capsule and EPM, Velocity of capsule, Rotation of camera mounted on capsule, Coordinates of two end points of stomach	
**Ensure**:	
Reward of every step	
**Initialization**:	
Capsule angular velocity ← 0, Capsule velocity ← 0;	
EPM angular velocity ← 0, EPM velocity ← 0;	
EPM and capsule position ← Initial position;	
End_1_, End_2_ ← Edge‐contour‐depth fusion module, Target end ← End_2_;	
Boundary reward ← 0;	
End reward ← 0, Total reward[] ← 0;	
Coverage reward[] ← 0, Track reward[] ← 0;	
**while** step ≤ 10000 **do**	
**Collect Observations**:	
Capsule velocity and rotation ← Unity;	
EPM position and rotation relative to the capsule ← Unity;	
End points position relative to the capsule ← Unity;	
**Get action from PPO**:	
EPM velocity ← Neural network;	
EPM angular velocity ← Neural network;	
EPM position ← EPM position + EPM velocity × Δt;	
EPM rotation ← EPM rotation + EPM angular velocity × Δt;	
Capsule position and rotation ← Dipole model;	
**Calculate reward**:	
Coverage diff ← |Current coverage – Previous coverage|;	
**if** Coverage diff > 0.03 then	
Coverage reward[step] ← Coverage diff × 20;	
**else**	
Coverage reward[step] ← −1;	
end if	
if |Capsule position − End2| ≤ 0.3 and Target end = = End2 then	
End reward ← 3;	
Target end ← End1;	
else if |Capsule position − End1| ≤ 0.3 and Target end = = End1 then	
End reward ← 3;	
Target end ← End2;	
end if	
Track reward[step] ← −|Capsule position − Target end| + End reward;	
if |Capsule position − EPM position| > 4 then	
Boundary reward ← −50;	
End episode	
end if	
Total reward[step] ← Coverage reward[step] + Track reward[step] + Boundary reward.	
**end while**

### 4.6 Animal Studies

In vivo experiment (Figure S18) was conducted on Yorkshire swine (n = 1, weight 30 kg), obtained from the Shandong Quality Inspection Center for Medical Devices. All experimental procedures and animal handling protocols were rigorously reviewed and approved by the Qilu Hospital of Shandong University. The ethical approval number is KYLL‐2022(ZM)‐017. Prior to the experiment, the animal was fasted for 12 h to ensure a clear gastric view. Anesthesia was induced via an intramuscular injection of ketamine (25 mg/kg) and subsequently maintained through continuous intravenous infusion of propofol (800 µg/kg/h) to ensure stable sedation throughout the navigation procedure. Vital signs were continuously monitored to maintain physiological stability.

## Conflicts of Interest

The authors declare no conflicts of interest.

## Supporting information




**Supporting File 1**: advs76024‐sup‐0001‐SuppMat.pdf.

**Supporting File 2**: advs76024‐sup‐0002‐MovieS1.mp4.

**Supporting File 3**: advs76024‐sup‐0003‐MovieS2.mp4.

## Data Availability

The main data supporting the results in this study are available within the paper and its supporting information.
