# Supplementary material for: Transferable Deep Reinforcement Learning With Edge‐Contour‐Depth Fusion for Autonomous Wireless Capsule Endoscopy Navigation
Source: Adv Sci (Weinh). 2026 Jun 12:e00008. Online ahead of print. doi: 10.1002/advs.202600008 (PMC13336913; doi:10.1002/advs.202600008)
Supplement: Supplementary file 1 — Supporting File 1: advs76024‐sup‐0001‐SuppMat.pdf. [file ADVS-9999-e00008-s003.pdf]

Supporting Information

Transferable Deep Reinforcement Learning with Edge-Contour-Depth Fusion for Autonomous Wireless Capsule Endoscopy Navigation

Haoxuan Wu[1†], Haitao Gao[2†], Qingyang Liu[1†], Sishen Yuan[1], Haiyang Fang[3], Mingwu Su[1], Baijia Liang[1], Yongzun Yang[1], Long Bai[1], Wenzhen Dong[1], Dihong Xie[1], Shijian Su[1], Jiewen Lai[1], Shing Shin Cheng[3], Zhen Li[4], Xiuli Zuo[4], Hongliang Ren[1*]*

This PDF file includes:

Notes S1 to S11

Figures S1 to S22

Table S1 to S12

References

Other Supplementary Material for this manuscript includes the following:

Movie S1. Robust transferability of AL-DRL-guided WCE navigation across diverse simulated anatomies.

Movie S2. Autonomous capsule endoscopy navigation within an ex-vivo pig stomach model by sim-to-real transfer.

Note S1. Virtual stomach modelling

A virtual gastric model was constructed using three-dimensional reconstruction from computed tomography (CT) imaging data obtained from the Cancer Imaging Archive (TCIA).

1. Data import and 3D reconstruction

The downloaded DICOM files were imported into InVesalius software, which converted the abdominal CT images into a comprehensive three-dimensional anatomical model including muscles, bones, tissues, and internal organs (Figure S1).

2. Segmentation and anatomical isolation

The exported STL file was imported into MeshLab, where non-gastric anatomical components—such as skeletal structures, muscle layers, liver, intestines, and other irrelevant tissues—were meticulously removed to isolate the stomach geometry.

3. Mesh optimization

To improve computational efficiency and surface quality, Quadric Edge Collapse Decimation was applied, simplifying the mesh topology and reducing vertex count. This process enhanced surface smoothness while substantially decreasing file size, ensuring suitability for downstream simulation tasks.

The finalized virtual stomach model is shown in Figure S2, providing an anatomically accurate and computationally efficient basis for the simulation environment.

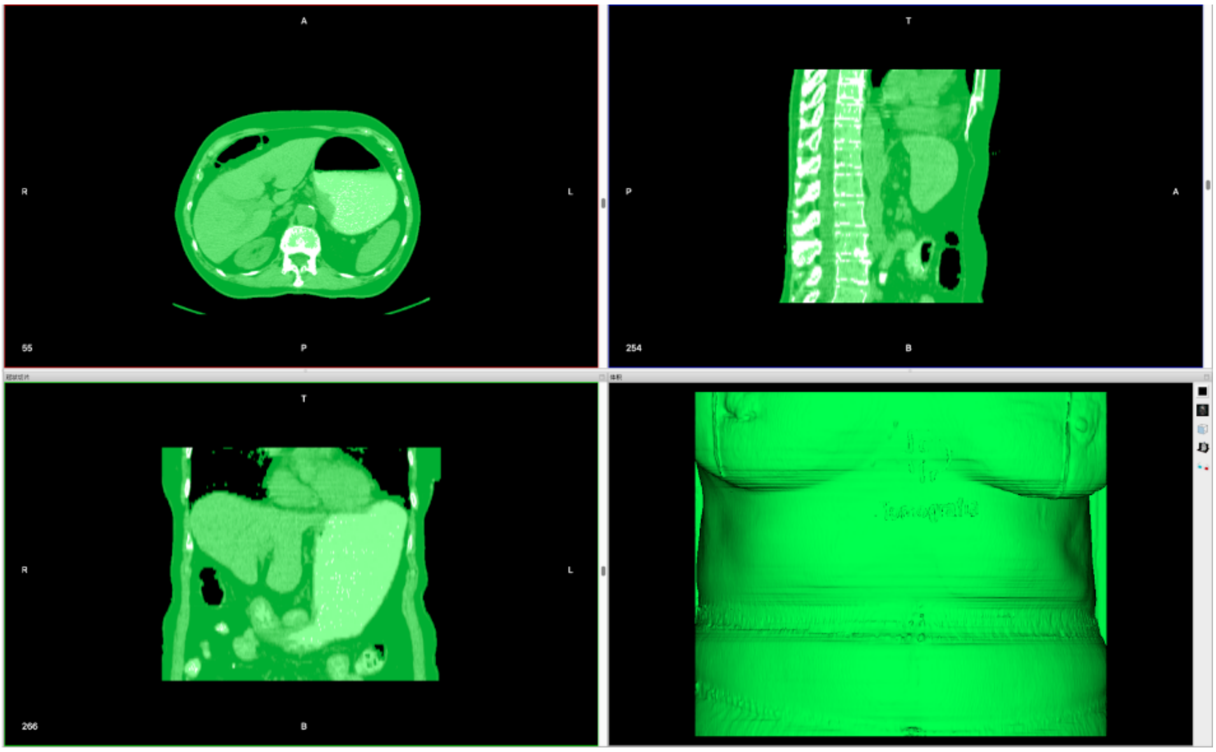

Figure S1. 3D reconstruction from CT scan images using InVesalius software.

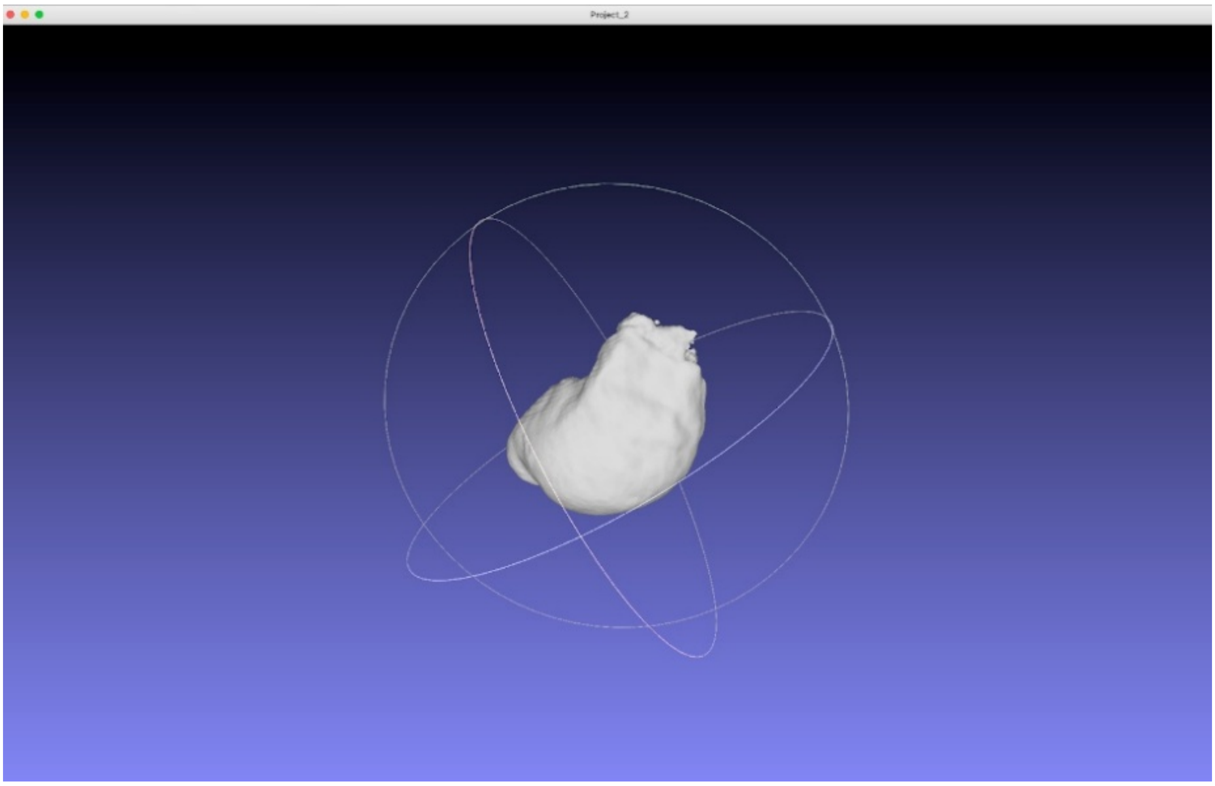

Figure S2. Final stomach model modified using MeshLab.

Note S2. DQN and SAC's performance with and without visual guidance

In the Transferability Characterization of the Offline AL-DRL Framework section, we quantitatively evaluated Proximal Policy Optimization (PPO)'s performance; here we present the corresponding results for Deep Q Network (DQN) and Soft Actor-Critic (SAC) under both visual-guidance and non-guidance settings. Compared with PPO, both DQN and SAC exhibited greater instability during training, with standard deviations of 14.36 and 25.54, respectively, versus 10.20 for PPO (Figure S3).

Navigation performance is illustrated in Figure S4, which shows coverage trajectories and performance metrics for PPO, SAC, and DQN in the same stomach environment. While SAC and DQN achieve comparable coverage ratios of approximately 90%, both require longer navigation times than PPO. In contrast, PPO consistently achieves higher coverage with greater time efficiency, highlighting its superior balance between exploration and exploitation as well as its policy robustness in this anatomical setting.

As discussed in the main text, DQN and SAC—similar to PPO—suffer marked performance degradation on unseen stomach models in the absence of visual guidance. Specifically, DQN attains an average coverage of 80.38% in the training stomach model but experiences reductions of 47.31%, 49.59%, 51.22%, 74.01%, 61.82%, 75.09%, and 64.22% across seven unseen stomach geometries (Figure S5). Likewise, SAC achieves an average training-model coverage of 90.54%, but its performance declines by 74.64%, 41.09%, 43.33%, 48.36%, 40.28%, 50.43%, and 47.79% on the same set of unseen models (Figure S6).

When visual guidance is incorporated, performance on unseen stomachs improves substantially due to the introduction of prior information—specifically, robust navigation trajectories derived from anatomical landmarks. Under this setting, DQN achieves an average coverage ratio of 84.45% across all eight stomachs, representing a 163.09% improvement over the baseline DQN without guidance (Table S1 and Figure S7), while SAC attains an average coverage of 86.91%, corresponding to a 172.04% improvement (Table S1 and Figure S8). These results confirm that visual guidance substantially enhances the transferability capability of both algorithms, mitigating the severe performance drop otherwise observed on unseen anatomical models.

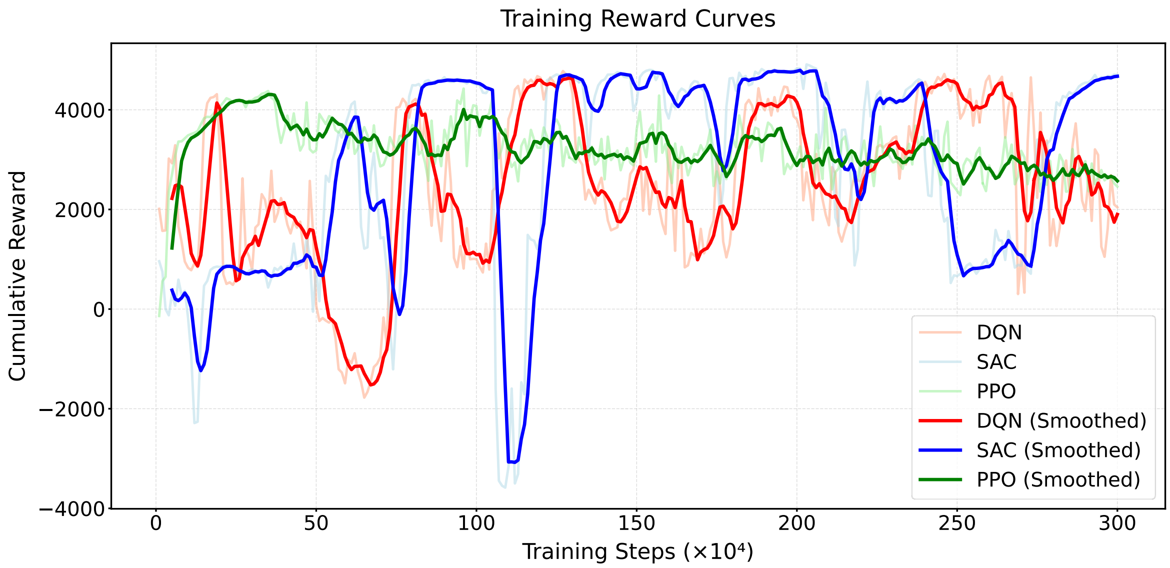

Figure S3. Training process of DRL algorithms.

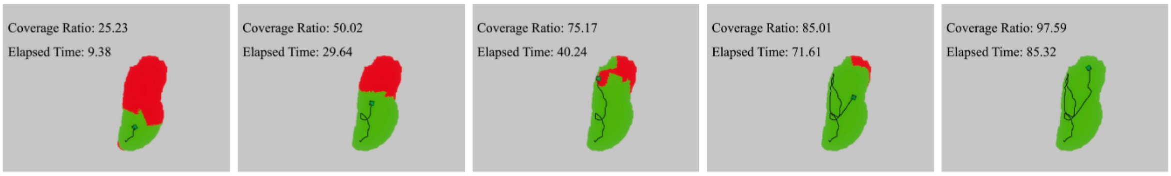

(a) PPO

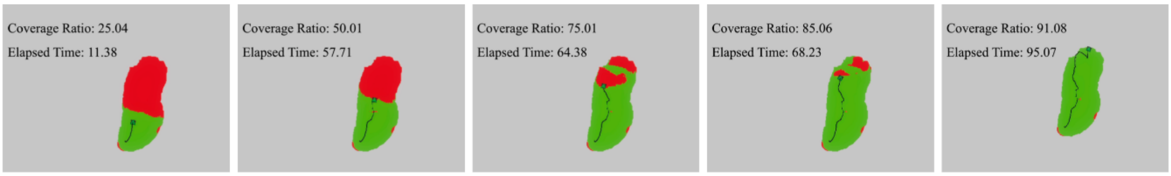

(b) SAC

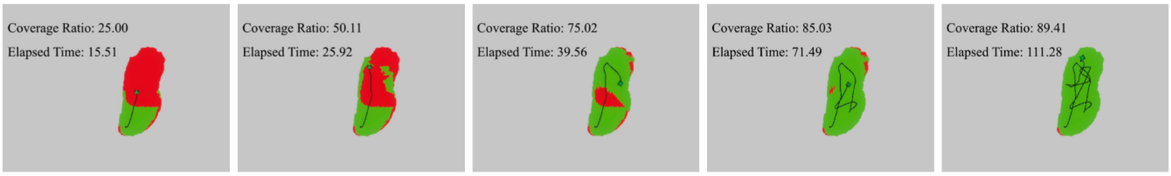

(c) DQN

Figure S4. Comparative performance of DRL algorithms on a single stomach model.

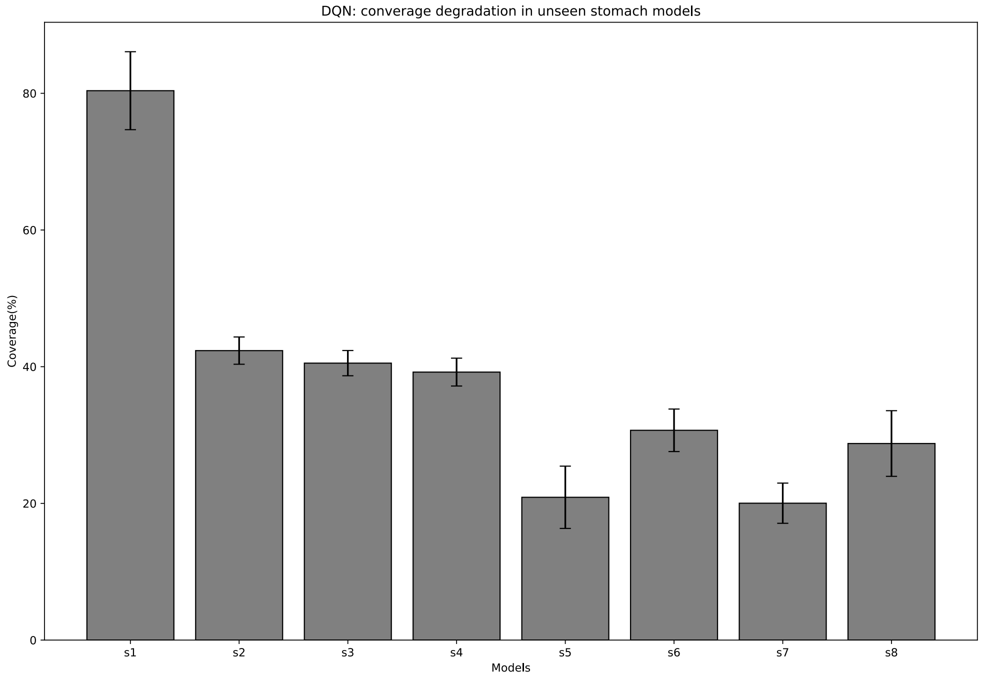

Figure S5. DQN: coverage degradation in unseen stomach models.

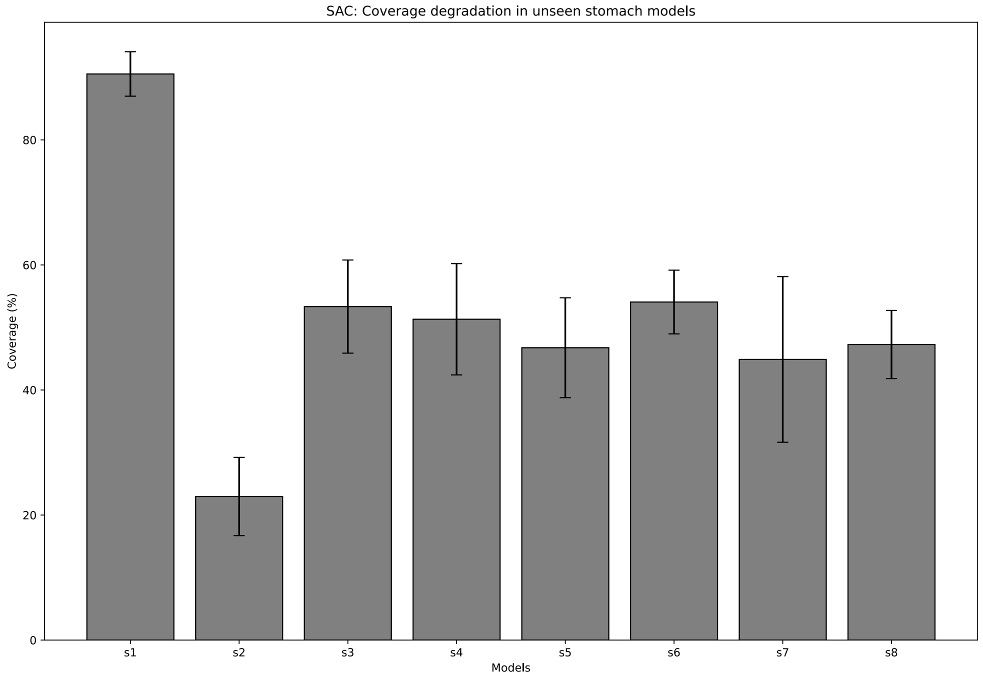

Figure S6. SAC: coverage degradation in unseen stomach models.

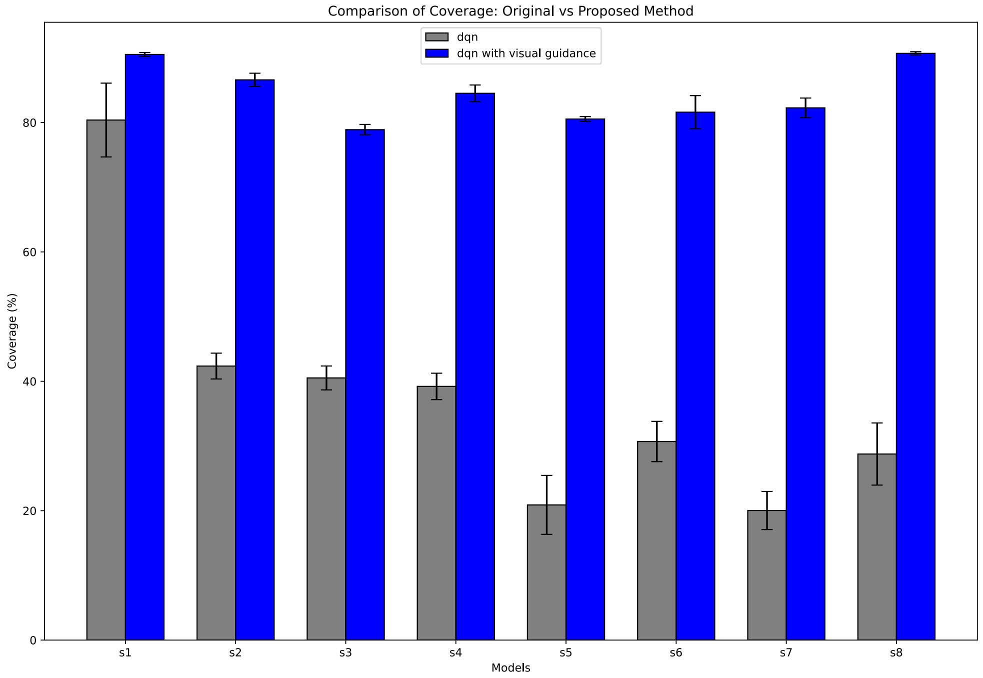

Figure S7. DQN: Coverage comparison between default and proposed method.

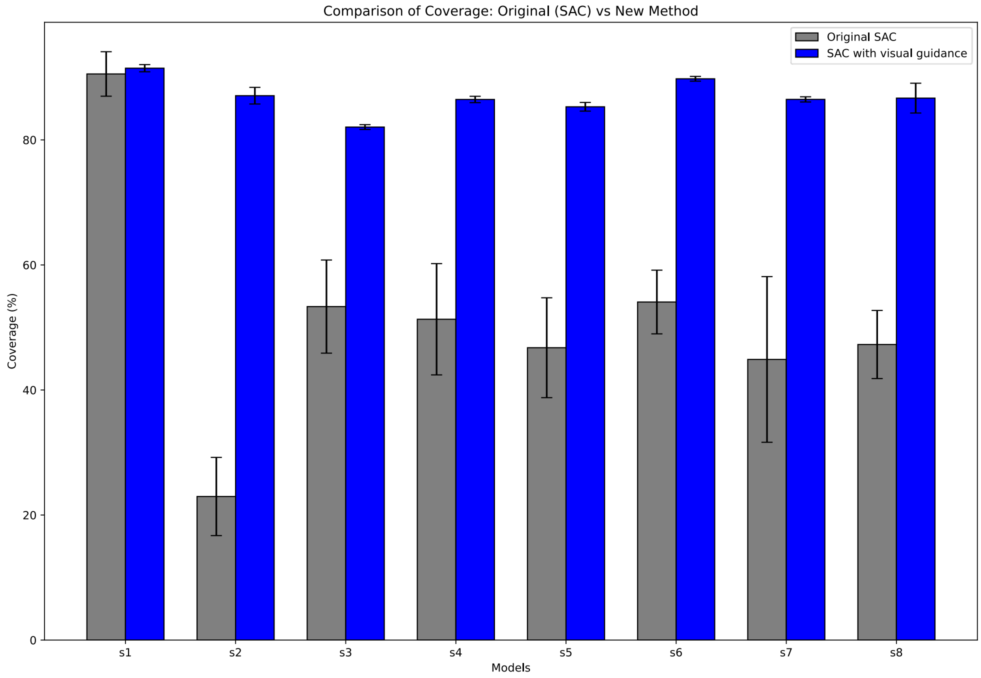

Figure S8. SAC: Coverage comparison between default and proposed method.

Table S1. Impact of landmark guidance on the performance of DQN and SAC

Algorithm	stomach1 (%)	stomach2 (%)	stomach3 (%)	stomach4 (%)	stomach5 (%)	stomach6 (%)	stomach7 (%)	stomach8 (%)
DQN	80.38	42.35	40.52	39.21	20.89	30.69	20.03	28.76
SAC	90.54	22.96	53.34	51.31	46.76	54.07	44.88	47.27
DQN (with visual guidance)	90.52 (12.61 ↑)	86.59 (104.46↑)	78.89 (94.70 ↑)	84.51 (115.53↑)	80.54 (285.51↑)	81.6 (165.84↑)	82.27 (310.78↑)	90.69 (215.32↑)
SAC (with visual guidance)	91.47 (13.79 ↑)	87.07 (105.58↑)	82.05 (102.49↑)	86.47 (120.52↑)	85.29 (308.26↑)	89.77 (192.47↑)	86.48 (331.81↑)	86.68 (201.39↑)

Note S3. Landmark selection criterion experiment

We conducted a verification experiment to evaluate the three proposed landmark selection criteria—universality, distinction, and navigation utility—across different gastric models. For universality, images of the fundus and pyloric antrum, as well as the cardia and pylorus, were collected from each stomach model. Circular edge detection achieved 100% accuracy in both the fundus/pyloric antrum and cardia/pylorus regions, demonstrating that these landmarks are universal, stable, and consistent across all models. In contrast, the angle of His/pyloric canal and the greater/lesser curvature also exhibited edge features, but these were inconsistent across images and provided little navigational value, resulting in a detection rate effectively considered to be 0.

In terms of distinction, the scaled edge intensity of the fundus/pyloric antrum averaged 1.00, significantly higher than the cardia/pylorus at 0.37, the greater/lesser curvature at 0.62, and the angle of His/pyloric canal at 0.58. Although the greater/lesser curvature and the angle of His/pyloric canal exhibited higher intensities than the cardia/pylorus, their edge patterns were inconsistent and unstable, even within the same stomach model. To further examine robustness, image perturbations—including scaling, 60° rotation, Gaussian noise, and lighting changes—were applied to simulate real-world navigation conditions. The fundus/pyloric antrum and cardia/pylorus remained detectable under scaling, rotation, and lighting changes, but noise rendered them undetectable. The greater/lesser curvature and angle of His/pyloric canal were undetectable under all applied transformations, as illustrated in Figure S9.

Regarding navigation utility, the AL-DRL framework achieved a global coverage of $97.3 \pm 2.0\%$ when using the fundus/pyloric antrum as landmarks, $76.72 \pm 0.94\%$ with the cardia/pylorus, and only $61.9 \pm 25.3\%$ and $58.23 \pm 21.35\%$ without effective landmark guidance—corresponding approximately to the use of greater/lesser curvature and angle of His/pyloric canal, respectively. To enable direct comparison among the four landmark types, all three metrics—detection rate, edge intensity, and global coverage—were normalized to a 0–1 scale. As shown in Figure S10, the fundus/pyloric antrum consistently outperformed the other three landmark types across all metrics, confirming their superiority as navigation markers in gastric environments.

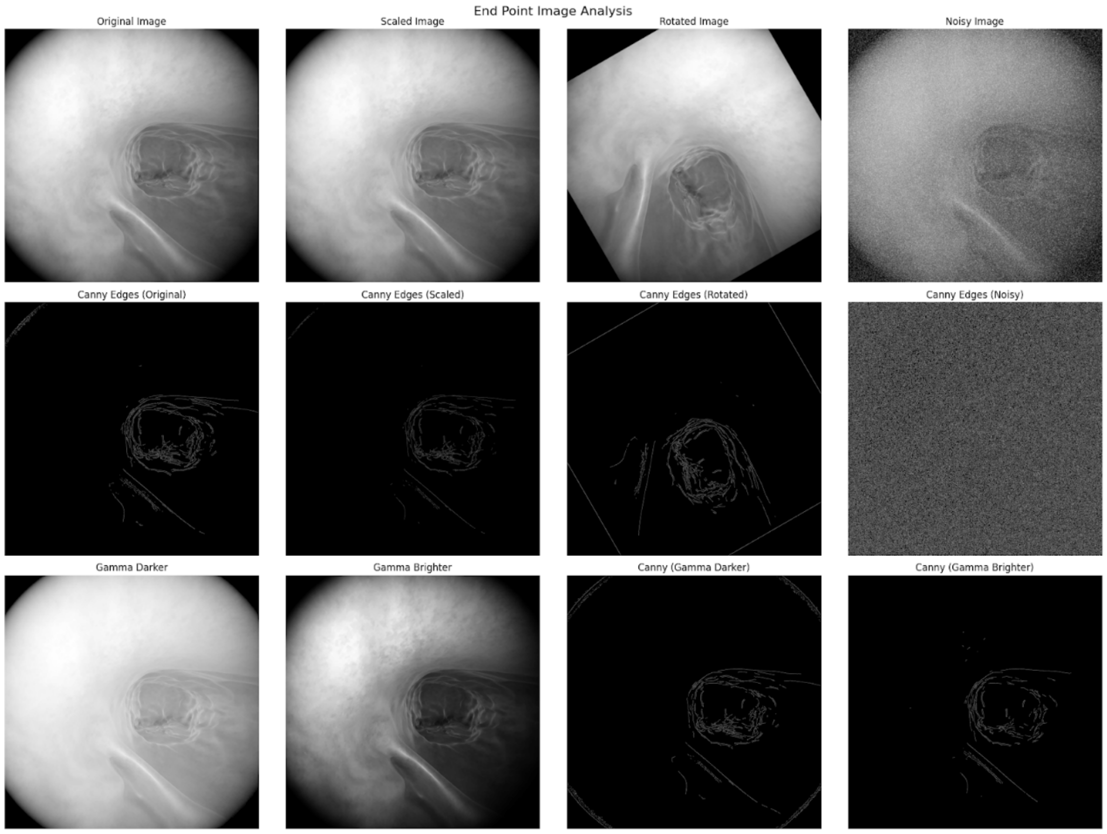
End Point Image Analysis
Original Image
Scaled Image
Rotated Image
Noisy Image
Canny Edges (Original)
Canny Edges (Scaled)
Canny Edges (Rotated)
Canny Edges (Noisy)
Gamma Darker
Gamma Brighter
Canny (Gamma Darker)
Canny (Gamma Brighter)

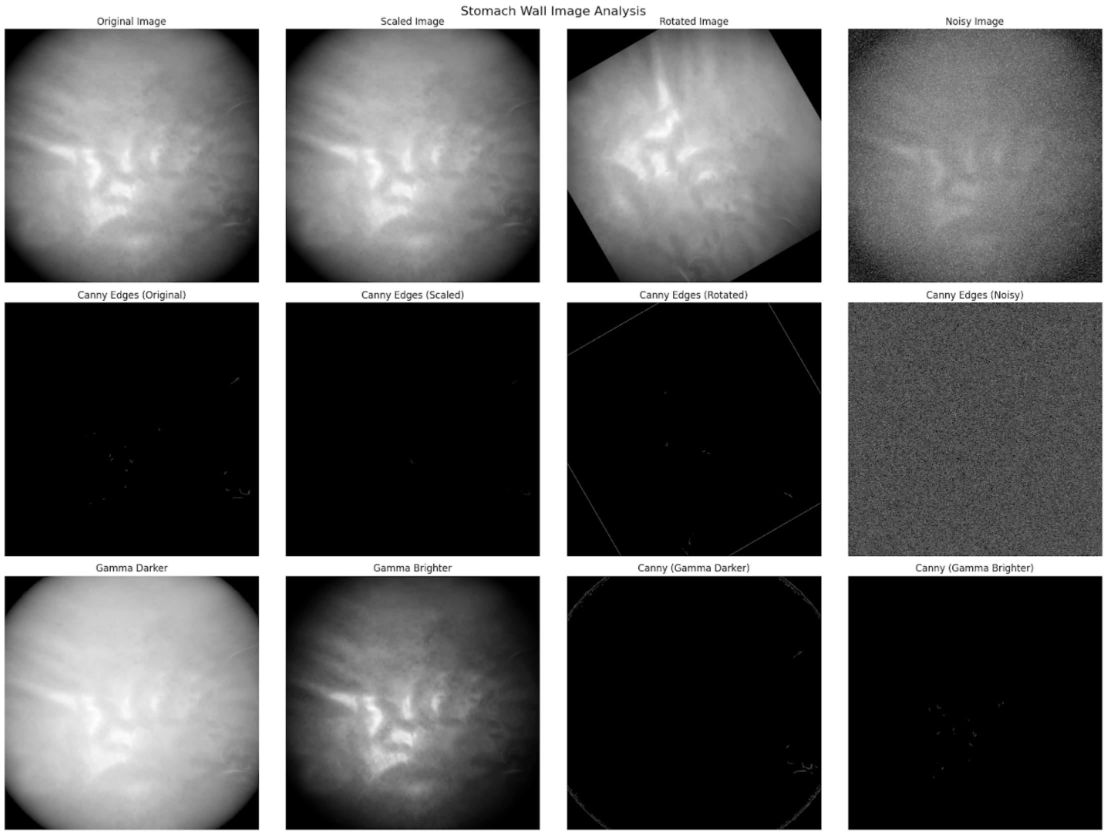
Stomach Wall Image Analysis
Original Image
Scaled Image
Rotated Image
Noisy Image
Canny Edges (Original)
Canny Edges (Scaled)
Canny Edges (Rotated)
Canny Edges (Noisy)
Gamma Darker
Gamma Brighter
Canny (Gamma Darker)
Canny (Gamma Brighter)

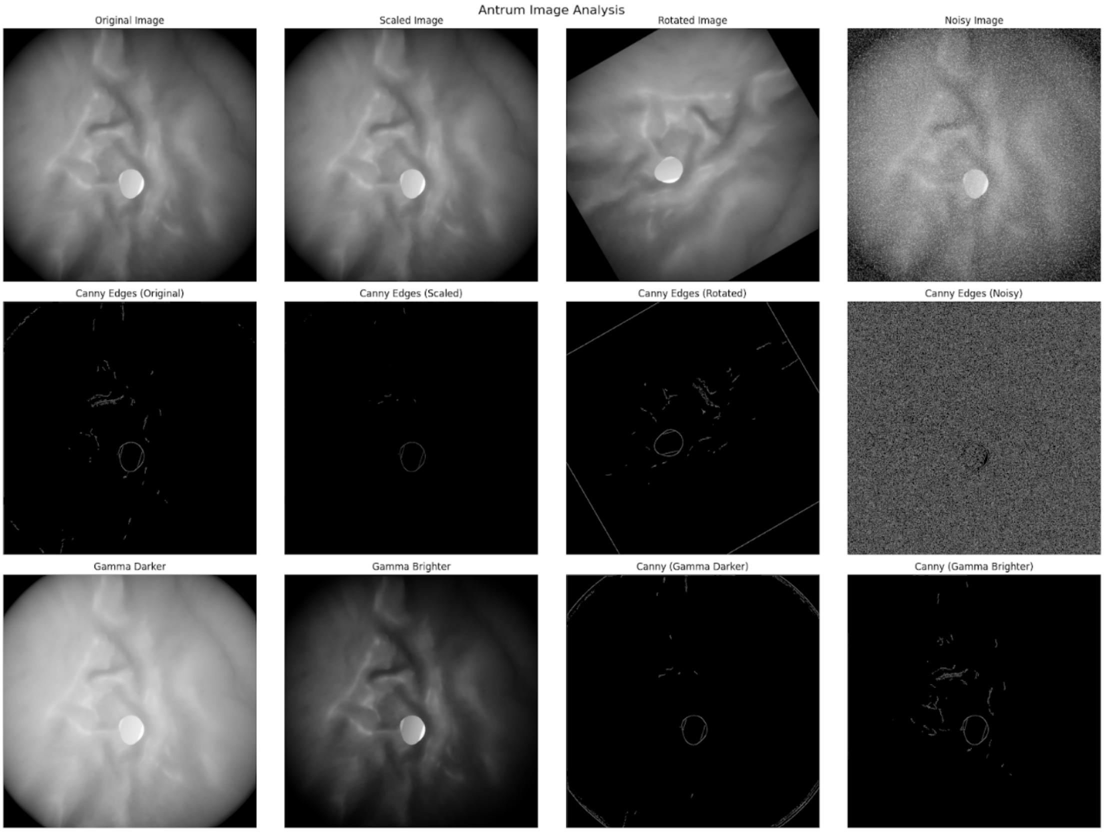

Figure S9. Detect the landmarks in different image distortions.

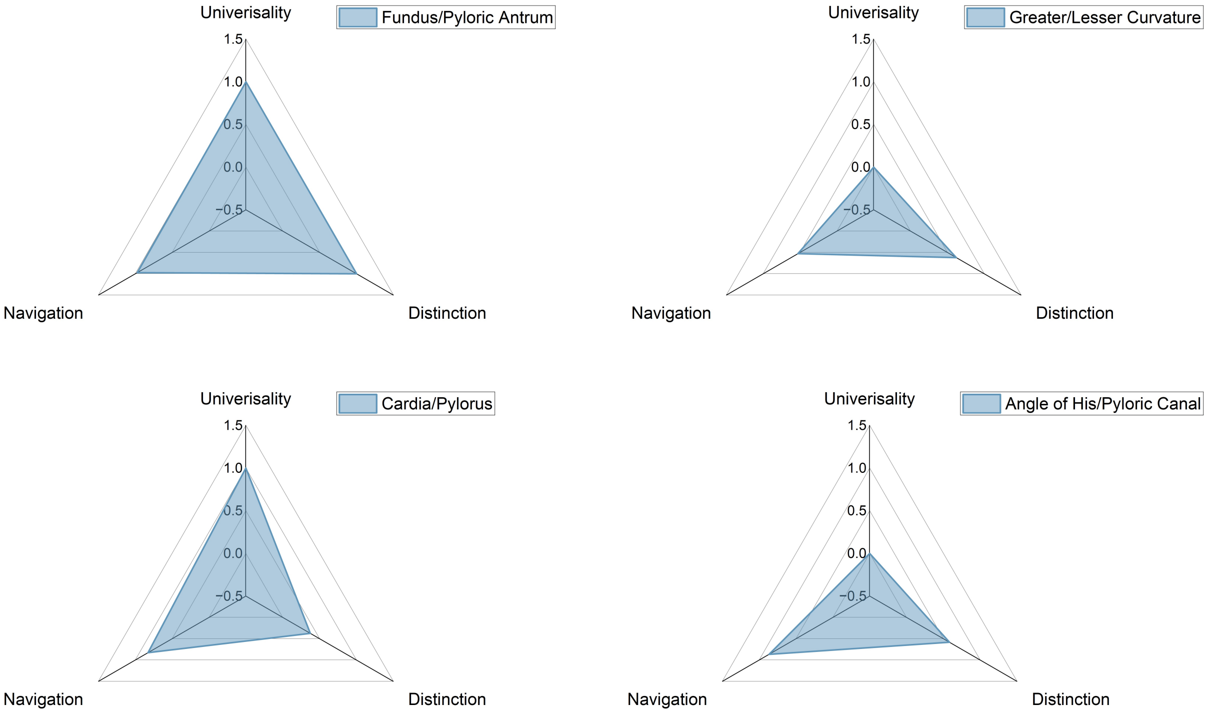

Figure S10. Performance of different landmarks.

Note S4. Statistical analysis of simulated stomach model

In the simulator, the stomach is represented using point clouds, enabling an accurate and detailed depiction of its geometric structure. Eight distinct stomach models were constructed, varying in both the number of points and the distribution of internal points, to reflect anatomical variability among patients (Figure S11).

To quantitatively evaluate similarity and variance among these models, we applied two complementary metrics: Chamfer distance to measure geometric similarity between stomach shapes, and voxel histogram similarity to assess differences in internal point distributions[1]. For each model, the 3D coordinates of its point cloud were extracted and normalized so that the centroid was positioned at the origin. These normalized datasets were then used to compute the two metrics.

As an example, Figure S12 presents a scatter plot comparing the geometries of stomach models 1 and 2, accompanied by histograms illustrating the internal point distributions of each model. Across all eight stomach models, the mean chamfer distance was 0.173, indicating high geometric similarity, whereas the mean voxel histogram similarity was only 0.184 (Figure S13), reflecting substantial variation in internal point distribution.

This analysis suggests that while patient stomachs often share comparable overall geometric envelopes, their internal anatomical configurations can differ markedly. Such differences may include variations in the size and arrangement of gastric folds, the relative positioning of the antrum and body, or the dimensions of the pyloric opening. These findings underscore the importance of developing adaptive navigation strategies capable of accommodating significant internal structural variability.

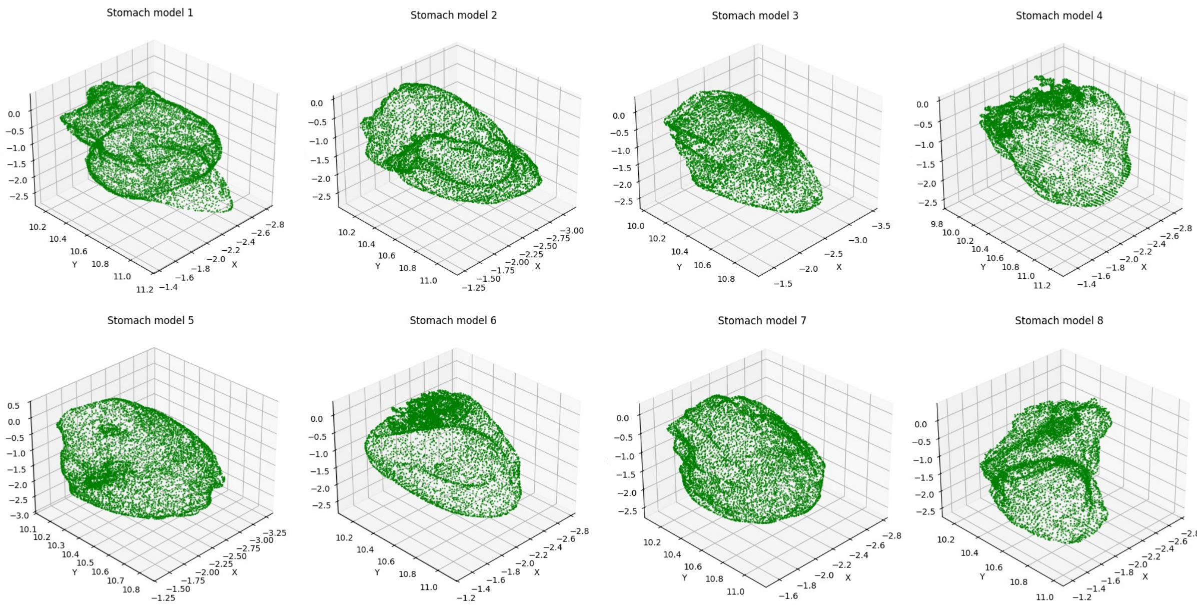

(a)

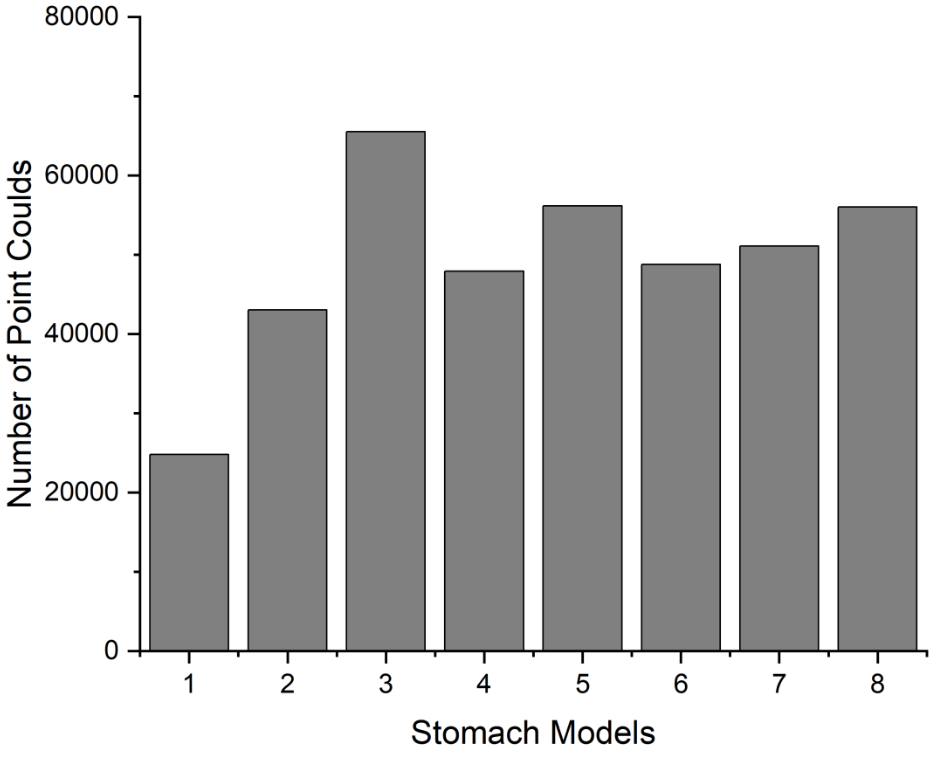

(b)

Figure S11. (a) Visualization of point cloud-based stomach model. (b) Number of point clouds in each stomach model.

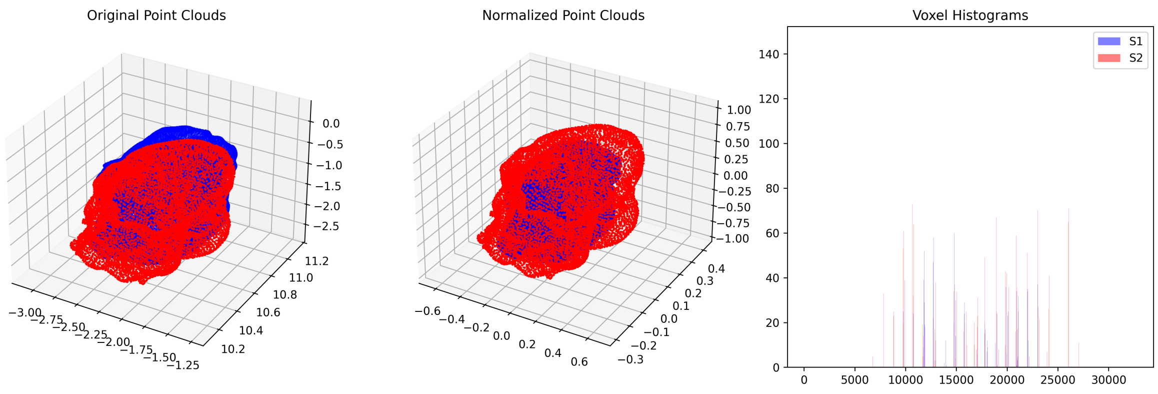

Figure S12. Point cloud geometrical distribution and internal data distribution difference in stomach model 1 and 2.

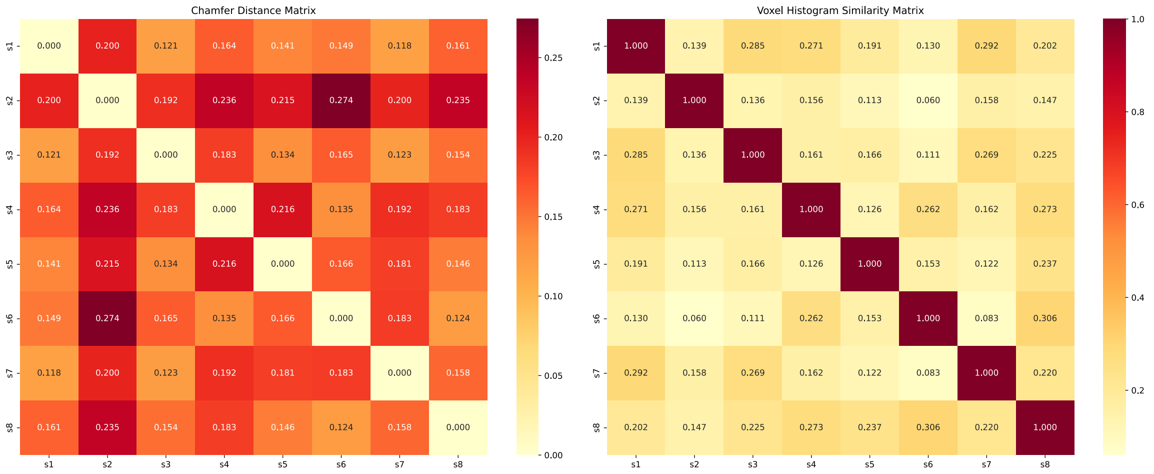

Figure S13. Distance matrix of charmer distance and voxel histogram similarity.

Note S5. Motion planning of the robotic arm

Conventional methods for navigating a wireless capsule endoscopy (WCE) equipped with an array of internal permanent magnet (IPM) via an external permanent magnet (EPM) manipulated by a robotic arm are typically model-based[2,3]. In such approaches, a magnetic dipole model is used to establish a linear mapping between the forces and torques acting on the WCE and the displacement and magnetic moment of the EPM[4,5]. The EPM's incremental translations and rotations are computed at each time step according to a predefined WCE trajectory, and transformed into joint-space commands via inverse kinematics[6,7]. These commands are applied as position control inputs to the robotic arm.

Although straightforward, this position-command control suffers from:

- Low achievable control frequency (due to iterative position updates and kinematic conversion steps)
- Limited robustness against disturbances in WCE manipulation
- Reduced smoothness in the EPM's motion trajectory

ADP-Based Velocity-Level Planning

To overcome these limitations, we adopt a model-free control scheme based on an Adaptive Dynamic Programming (ADP) robust tracking controller. Here:

- The optimal global scanning path of the WCE is first generated in simulation.
- The controller tracks this reference path at the velocity level, directly producing six-dimensional end-effector velocity commands for the EPM.
- This velocity-level planning results in smoother and more dynamically feasible EPM trajectories than position-level control.

Simulation-to-Physical Coordinate Alignment

Before execution, the WCE path (generated in the stomach phantom coordinate system {P} within the simulation) must be transformed to the robot base coordinate frame {B} used in the laboratory:

1. The 3D digital model of the gastric phantom is registered to the robot base frame {B} based on the phantom's known fixed position and orientation relative to the robot base.
2. This yields a homogeneous transformation matrix T_P^B between {P} and {B}.
3. Each path point P^P from the simulation is transformed using:

$$P^B = T_P^B P^P \tag{1}$$

The resulting real-world trajectory P^B is fed to the ADP controller, which outputs the corresponding six degrees of freedom (6-DOF) end-effector velocity vector $\dot{\boldsymbol{x}}$.

Inverse Kinematics and Velocity Mapping

The velocity $\dot{\boldsymbol{x}}$ in Cartesian space must be converted to joint-space velocities $\dot{\boldsymbol{q}}$ before being sent to the robot's servo controller[8]. This mapping uses the robot arm Jacobian $J(\boldsymbol{q})$:

$$\dot{q} = J^{\dagger}(q)\dot{x} + \left(I - J^{\dagger}(q)J(q)\right)\dot{q}_0 \tag{2}$$

where

$J^{\dagger}$ - Moore–Penrose pseudoinverse of J;

$\dot{q}_0$ - null-space velocity for secondary objectives (e.g., joint-limit avoidance);

I - identity matrix.

This formulation enables redundancy resolution and smooth joint-limit avoidance during navigation.

Velocity Filtering and Safety Constraints

The raw joint velocity sequence from the ADP controller[9] can contain spikes or discontinuities caused by environmental disturbances or numerical artifacts, which may induce excessive joint accelerations, risking both tracking instability and mechanical safety.

1. Threshold Filtering

A per-joint velocity limit is enforced:

$$\dot{q}_i \in [-v_{max}, v_{max}] \tag{3}$$

where $\dot{q}_i$ is the i-th joint velocity, and v_{max} is the manufacturer-specified maximum velocity for that joint. Any out-of-range values are replaced by the mean of their immediate neighbors, preserving sequence length.

2. Butterworth Low-Pass Filtering

Even after clipping, high-frequency oscillations can remain. To suppress these without distorting the intended motion trend:

- A second-order Butterworth low-pass filter[10] ($N = 2$) is applied to each joint's velocity time series.
- High-frequency components correspond to abrupt velocity jumps (undesirable), while low-frequency components correspond to the smooth motion envelope (desirable).
- The normalized cutoff frequency is:

$$W_n = 2\frac{f_c}{f_s} \tag{4}$$

where

W_n - normalized cutoff frequency;

f_s - velocity command sampling rate;

f_c - chosen cutoff frequency (below Nyquist frequency $\frac{f_s}{2}$)

The filter parameters are chosen to eliminate acceleration spikes while retaining trajectory fidelity.

Execution in Robot Controller

After threshold limiting and low-pass smoothing, the joint-space velocity sequence is sent to the robot arm controller via the MoveIt API.

Note S6. Force analysis for capsule endoscope

To minimize the reality gap in transferring the proposed navigation framework from simulation to real-world applications, we performed a detailed force analysis of the capsule endoscope, as illustrated in Figure S14. The capsule's motion within the gastric environment is governed primarily by:

1. Gravitational force;
2. Frictional interactions between the capsule surface and gastric mucosa;
3. Magnetic forces and torques generated by the interaction between the EPM and the IPM.

Simulation Environment

All force interactions were modelled in the Unity platform using the NVIDIA PhysX physics engine, which supports both discrete and continuous collision detection (CCD). CCD ensures stable and precise modelling of contact dynamics even at high velocities. On mid-range computing hardware, the physics computation step remains below 2 ms per frame for moderately complex scenes, enabling accurate real-time approximation of continuous physical behaviors.

Parameter Configuration

To closely replicate realistic conditions, the following parameters were explicitly set in simulation:

- Gravitational acceleration: $g=9.81\ m/s^2$
- Friction coefficient (gastric mucosa, smooth areas): $\mu=0.10$
- Friction coefficient (capsule surface): $\mu=0.50$
- Magnetic moment (EPM): $119.36\ A{\cdot}m^2$
- Magnetic moment (IPM): $0.55\ A{\cdot}m^2$

Given the magnetic field's inverse cubic decay with distance ($B \propto 1/r^3$), capsule motion is highly sensitive to positional variations. Consequently, free-floating motion within the gastric lumen is rarely observed; instead, both simulations and physical experiments consistently show that the capsule predominantly navigates along the gastric wall. This makes accurate friction modelling a critical factor for reliable sim-to-real transfer.

Friction Modelling and Physiological Variability

To account for anatomical and physiological variability:

- Spatially varying friction coefficients were assigned across gastric regions:
  - Lower values for smoother mucosal surfaces
  - Higher values for regions with pronounced folds or irregularities

- Stochastic perturbations were applied to the friction coefficients at runtime to emulate transient variations caused by gastric peristalsis, including dynamic changes in surface topography and mucosal fold geometry.

Impact on Sim-to-Real Transfer

By integrating realistic force modelling, region-dependent friction maps, and stochastic perturbations, the simulation environment captures key mechanical interactions affecting capsule motion. This reduces discrepancies between simulated and real-world capsule behavior, improving the transferability and robustness of navigation strategies.

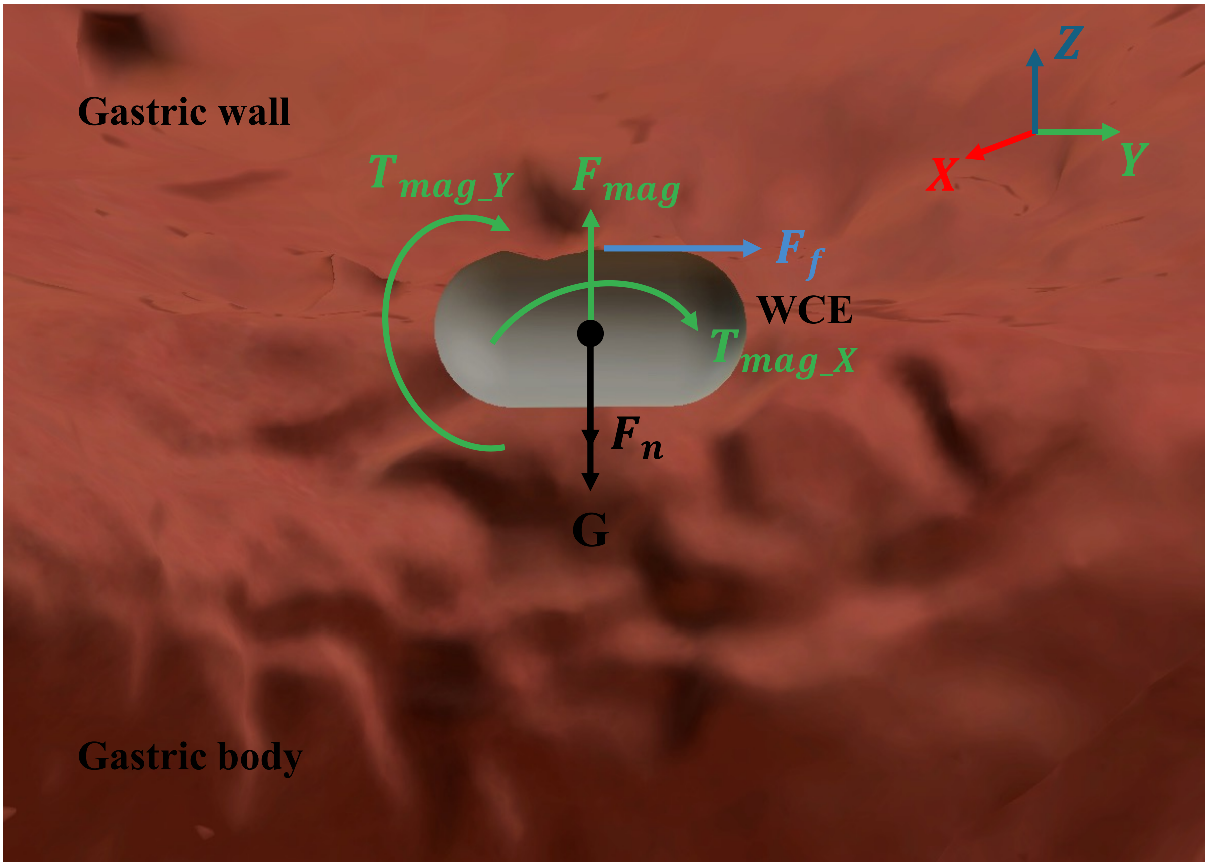

Figure S14. Force analysis of WCE during intragastric navigation.

Note S7. Low-cost capsule design for flexible 5-DOF magnetic control

The custom-built capsule endoscope measured 12 mm in diameter and 35 mm in length, integrating an OV9734 fiber-optic imaging module (1280 × 720 pixels at 30 fps; 102° field of view) and a specially engineered IPM array. The IPM array comprised four groups of NdFeB magnetic poles arranged with orthogonally anisotropic magnetic moment distributions (Figure S15), providing optimal controllability across five degrees of freedom. Compared to a single monolithic magnet, the distributed array of four axially magnetized poles is selected for structural integration and manufacturing feasibility. This configuration preserves the capsule's central longitudinal axis for the imaging sensor and optical path, avoiding the obstruction a monolithic magnet would cause. Furthermore, a single axially magnetized annular magnet conforming to these specific dimensions is technically challenging to fabricate and difficult to source commercially. This distributed design offers a practical, cost-effective integration strategy that maintains standard clinical capsule dimensions without compromising the internal hardware layout.

To ensure that the tether does not interfere with capsule motion during gastric scanning, we performed a finite element mechanical analysis using ABAQUS (Figure S16). In the simulation, the capsule was modelled as a rigid body, and the tether was represented as a composite structure consisting of a copper core wire (Young's modulus E =110 GPa) surrounded by a polyurethane sheath (E =0.1 GPa), with an overall diameter of 2 mm. Simulation results indicated that the effective combined Young's modulus of the tether was approximately 4 GPa. Under ±180° torsional deformation, the maximum torque generated by the tether was 0.04 N·mm, which is only about 10% of the magnetic torque (0.35 N·mm) exerted by the EPM on the capsule.

To empirically validate the operational range and the distance-dependent dominance of magnetic actuation, we performed torque-distance characterization using an ATI Nano17 6-axis force/torque sensor (Figure S17a). Measurements conducted across a distance range of 25 mm to 125 mm demonstrate that the magnetic torque remains consistently above the 0.04 N·mm resistive threshold of the tether throughout the entire workspace (Figure S17b). Notably, the system maintains the standardized working torque of 0.35 N·mm at a distance of approximately 90 mm, which is sufficient for robust maneuverability within the volume of a distended gastric lumen. This experimental data establishes a quantitative boundary for the system's clinical applicability, confirming that magnetic control remains the primary governing factor over mechanical tether resistance across all practical operational distances.

To validate these findings, in-vivo experiments were conducted in a live porcine model (Figure S18). The tethered capsule exhibited flexible bending at both 180° and 360° without noticeable restriction, confirming that the tether did not impede manoeuvrability within the stomach. The observed motion characteristics closely matched those of WCE, further supporting the conclusion that the tether has a negligible influence on capsule performance. Concurrently, the distinct visualization of gastric anatomy achieved under manual control substantiates the potential of handheld magnetic actuation as a viable, low-cost approach for portable endoscopic examinations.

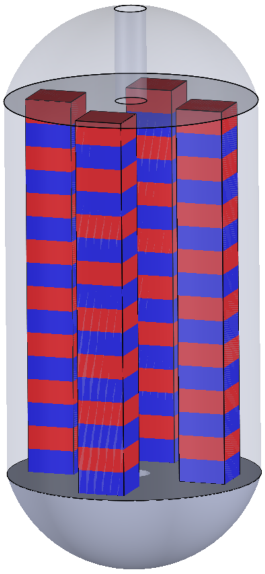

(a)

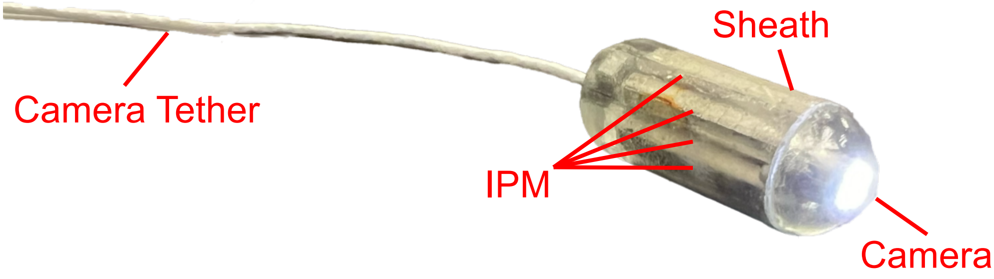

(b)

Figure S15. Capsule endoscope with designed IPM array and real-time visual monitoring.

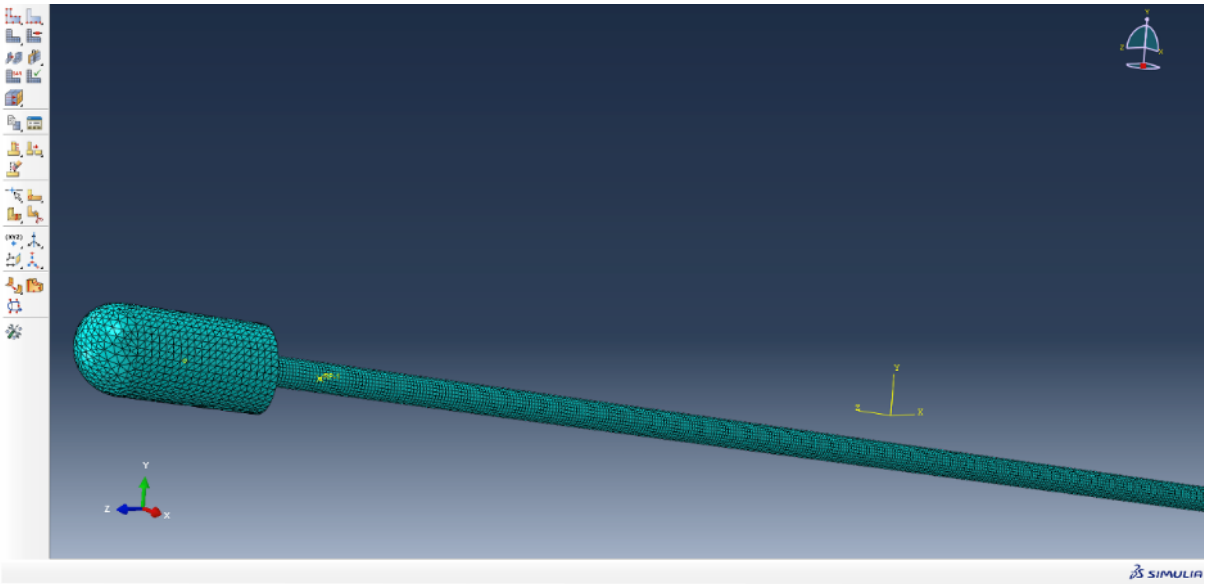

Figure S16. Finite element mechanical analysis of tethered capsule using Abaqus software.

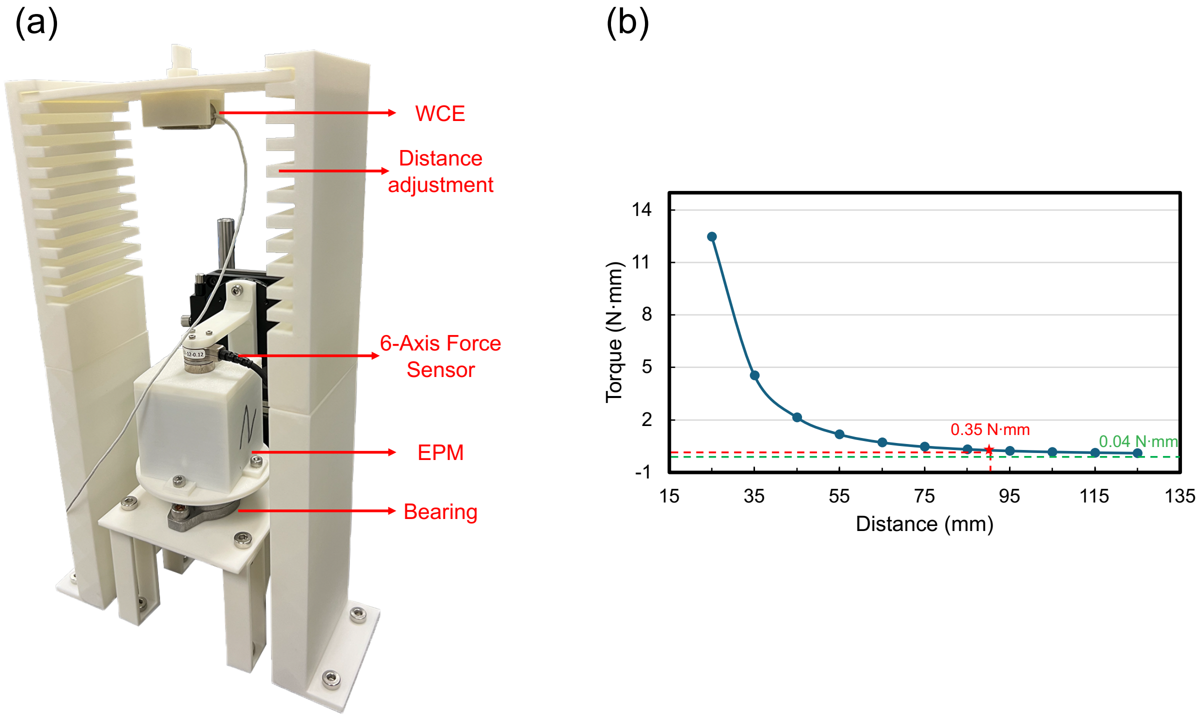

Figure S17. Experimental characterization of magnetic torque versus distance. (a) Experiment setup. (b) Experimental results highlighting the 0.04 N·mm tether resistance threshold and the 0.35 N·mm standardized working benchmark across the 25–125 mm range.

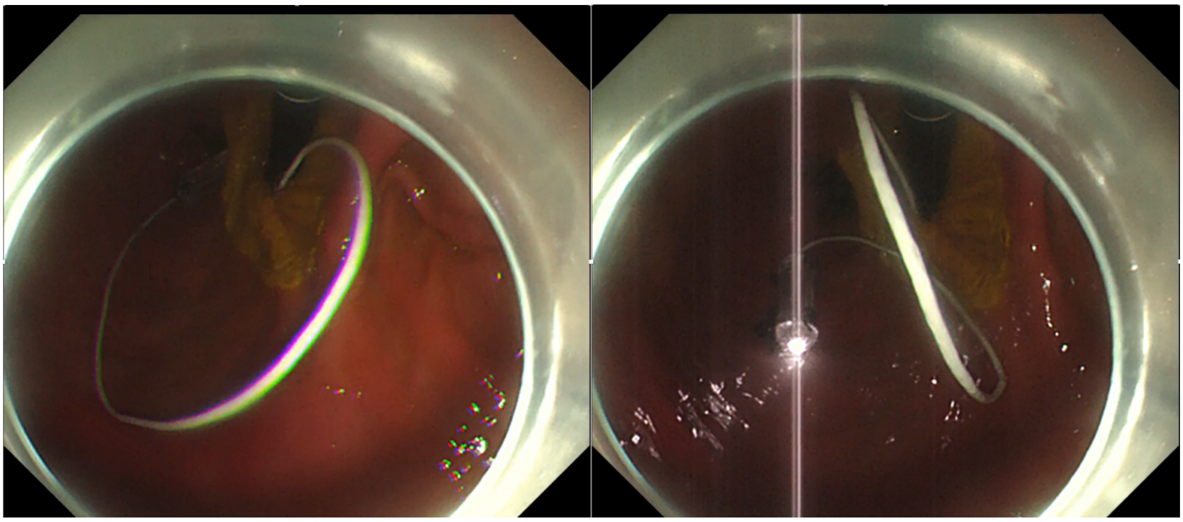

Figure S18. In vivo validation of tethered capsule manoeuvrability and handheld magnetic control feasibility. The images depict the tethered capsule undergoing flexible bending at 180° and 360° within the porcine stomach. These observations confirm that the low-modulus tether exerts negligible influence on the capsule’s motion dynamics, allowing for unrestricted manoeuvrability. Furthermore, the successful capture of clear gastric anatomical structures validates the practical feasibility of performing effective gastric scanning using more portable and low-cost handheld magnetic actuation.

Note S8. Magnetic localization algorithm for the magnetic sensor array

Accurate real-time localization of the capsule pose was achieved using AMagPoseNet[11], operating on a 5 × 5 magnetometer array (LIS3MDL, STMicroelectronics; ±1200 μT range), as shown in Figure S19. The 25 sensors were evenly soldered onto a printed circuit board at 40 mm intervals. Sensor data were acquired via an STM32H743 microcontroller and transmitted to a PC for processing.

AMagPoseNet employs a dual-domain few-shot learning framework. Unlike conventional models that rely solely on large amounts of real-world training data, this approach incorporates a prior physical model of the magnet's magnetic dipole field to assist in learning the mapping between magnetic induction intensity and 6-DOF pose. This reduces the required real-world sample size to only 780 training samples, significantly lowering data collection costs.

The AMagPoseNet architecture consists of two cascaded sub-networks:

- PoseNet: A synthetic dataset was first generated using a Biot–Savart–based magnetic field model, and PoseNet was trained to regress the 6-DOF pose from simulated magnetic measurements, with sine–cosine-encoded Euler angles to address magnetic field symmetry.
- CaliNet: To mitigate sensor noise and domain shift, this calibration network was trained using a small real-world dataset to map measured signals into the model domain.

During inference, real-time measurements were first corrected by CaliNet, then passed to PoseNet, yielding accurate (1.87 ± 1.14 mm, 1.89 ± 0.81°) and low-latency (2.08 ± 0.02 ms) six-DOF localization without iterative optimization.

Compensation for EPM–IPM Coupling

A critical challenge is suppressing localization interference from the EPM used for actuation. We address this via a two-pronged approach. The primary method is active compensation: the EPM's 6-DOF pose is tracked in real-time by deriving the robot's forward kinematics from its current joint states. This real-time pose is used in a magnetic dipole model (Eq. 7) to compute the EPM's interference field, which is then subtracted from the sensor array's total measurements to isolate the pure signal from the capsule's internal magnet. Calibration of the robotic arm, localization board, and EPM reference frames ensures accurate frame alignment for this calculation.

This active compensation is supplemented by a passive physical principle. The EPM is modelled as an ideal magnetic dipole[12], with its field magnitude $\boldsymbol{B}(r)$ decaying rapidly with the cube of the distance $(\frac{1}{\boldsymbol{r}^3})$

$$\boldsymbol{B}(r) = \frac{\left(\frac{\mu_0}{4\pi}\right)[3(\boldsymbol{m}\overline{\boldsymbol{r}})\overline{\boldsymbol{r}} - \boldsymbol{m}]}{\boldsymbol{r}^3} \tag{5}$$

where $\boldsymbol{m}$ is the dipole moment, $\overline{\boldsymbol{r}}$ is the unit vector from the dipole to the field point, and μ_0 is the vacuum permeability. Because the EPM is placed significantly farther from the sensor array than the IPM, its measured field is inherently much weaker, further aiding in the suppression of EPM interference.

Magnetic Field Modelling

During pose measurement, the system acquires the spatial distribution of magnetic induction vectors via a 5×5 magnetic sensor array (LIS3MDL, STMicroelectronics). According to the Biot–Savart law, the magnetic induction intensity $d\boldsymbol{B_l}$ generated by a surface current source I at any point in space can be expressed as:

$$B_c = \int_{-r}^{r}\int_{ab}^{d} B_l + \int_{-r}^{r}\int_{bc}^{d} B_l + \int_{-r}^{r}\int_{cd}^{d} B_l + \int_{-r}^{r}\int_{da}^{d} B_l \qquad (6)$$

where ab, bc, cd, da represent magnetic induction contributions along the four edges of a rectangular differential current element, and r is the ring magnet radius.

For a ring magnet, the superposition principle can be applied: the net magnetic induction at any point p_i in space is the difference between the field of a solid cylinder with outer radius r_1 and that of a solid cylinder with inner radius r_2. Thus:

$$B_a = f_c(p_i\,, r_1) - f_c(p_i\,, r_2) \qquad (7)$$

where f_c denotes the closed-form field expression for B_c, computed here using Simpson's numerical integration method[13]. This computed B_a field, expressed in the magnet's local coordinate frame, is then transformed into the magnetic sensor array's coordinate frame $\{s\}$ for localization, whose origin is defined at the geometric center of the array PCB. For the i-th sensor, the transformed magnetic induction vector is modelled as:

$$B_i = F(p_i^s\,, v)\,, (i = 1, 2, \cdots, N) \qquad (8)$$

where p_i^s is the position of sensor i in {s} and v denotes the pose parameters of the magnet.

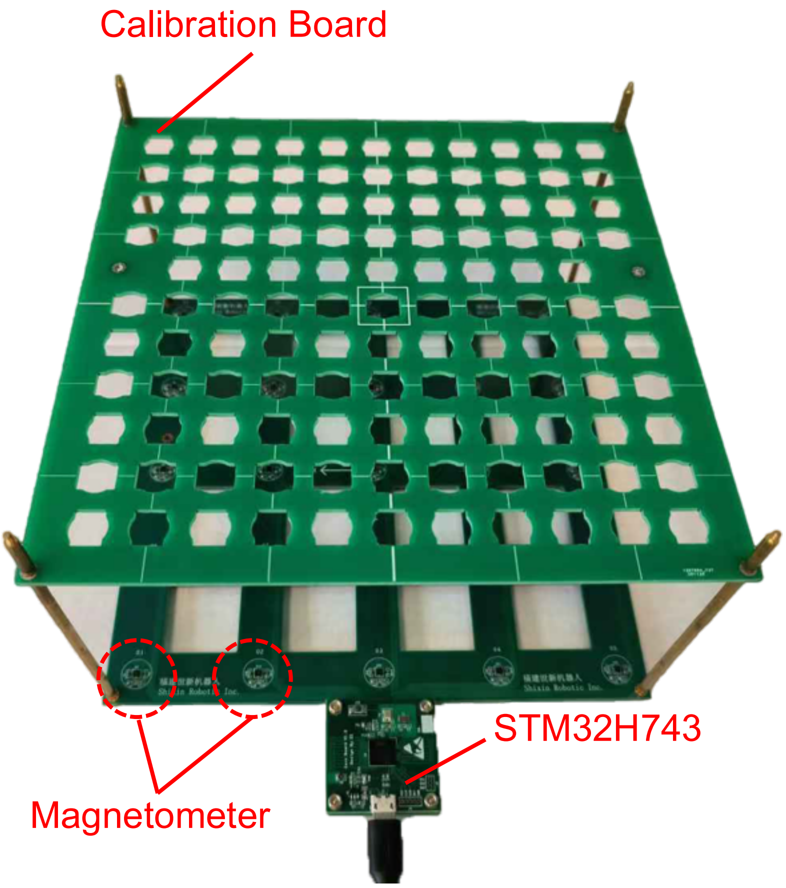

Figure S19. Magnetic localization board.

Note S9. Calibration of the hardware system

When driving the wireless capsule endoscope (WCE) inside the body by manipulating an EPM mounted on the robotic arm's end-effector, real-time localization of both the EPM and the WCE is essential[14,15]. The hardware localization architecture proposed in this study integrates a magnetic sensing subsystem for IPM pose estimation. The outputs of these subsystems are transformed into a common world coordinate frame to enable coordinated control. To ensure accuracy, each subsystem must be calibrated before use, and their coordinate frames must be registered.

Tracking of the EPM

The real-time pose of the EPM, rigidly attached to the end-effector, is derived from the robot's forward kinematics[16] within the base frame {B}.

- Physical setup: The EPM is defined as a rigid body fixed to the end-effector, with its pose in the base frame {B} determined from the robot's current joint states.
- Pose acquisition: The acquired information consists of the homogeneous transformation matrix $\mathrm{T_B^M}$, relating the EPM frame {M} to the base frame {B}.

To relate the EPM pose to the robotic arm's base frame {B}, an eye-to-hand calibration is performed using the easy_handeye package:

$$P^B = T_M^B P^M \tag{9}$$

where

$\{M\}$ - coordinate frame fixed to the EPM;

T_B^M - transformation from arm base to EPM frame (from forward kinematics);

Magnetic Sensing of the IPM

The IPM, embedded in the WCE, is invisible when inside the ex-vivo pig stomach. Its localization is obtained using a magnetic tracking system (MTS):

- Sensor array: A 5×5 magnetometer grid (LIS3MDL, STMicroelectronics) defines the sensor frame {S};
- Pose estimation: The AMagPoseNet neural network estimates IPM pose from the measured magnetic field vectors in real-time within the array's working volume.

The interference to localization accuracy arising from the magnetic field coupling between the IPM and the EPM is eliminated by leveraging the real-time EPM pose relative to the Magnetic Tracking System (MTS) to calculate and subtract the EPM's contribution to the measured field.

Registration of the Magnetic Array to the World Frame

To unify IPM and EPM localization results:

1. A 3D model of the magnetic array is registered to the robotic arm's base frame.
2. Landmark-based registration yields the transformation matrix T_S^B from the sensor frame {S} to the base frame {B}.

Given the IPM position P^S in {S}, its world frame coordinates are computed as:

$$P^B = T_S^B P^S \tag{10}$$

Unified Pose Data for Control

With both:

- EPM pose from forward kinematics
- IPM pose from magnetic sensing

expressed in the same base coordinate frame {B}, the control system can compute the instantaneous spatial relationship between EPM and IPM. This unified data is then used to plan and execute robotic arm motion for precise WCE navigation.

Note S10. Feature extraction methods comparison

To determine the optimal combination of edge detection and feature matching algorithms for robust landmark identification, we conducted a comprehensive comparison of traditional image processing techniques, implemented using the OpenCV library. Feature extraction was divided into two stages: (1) edge detection and (2) feature matching. Eight edge detection algorithms—Canny[17], Sobel, Laplacian, Prewitt, Scharr, morphological gradient, and Gabor—were evaluated in combination with three feature matching methods: contour matching[18], histogram of oriented gradients (HoG)[19], and structural similarity index measure (SSIM)[20].

For evaluation, three images were collected from each of eight stomach models: two depicting the endpoints of the stomach body and one showing a wall along the stomach's curvature. The task was to match the second endpoint image using the first endpoint image as a template, with the wall image serving as a distractor. The primary performance metric was the difference in match scores between the target (second endpoint) and the distractor (wall). A larger score difference indicates a stronger ability to distinguish the correct match, enabling a straightforward threshold for accurate identification.

All possible combinations of edge detection and feature matching methods were tested across the eight stomach models. For each experiment, the match score difference between the target and distractor was computed. The detailed results are presented in Tables S3–S10. To facilitate interpretation, the experimental data were further summarized into two structured tables—Table S11 for edge detection methods and Table S12 for feature matching methods—highlighting the relative performance of each approach in correctly identifying targets.

Quantitative analysis revealed that the Canny edge detector combined with contour matching consistently produced the largest similarity differences between the target and distractor, indicating superior robustness in endpoint recognition. As illustrated in Figure S20 and Figure S21, this combination achieved optimal matching performance for identifying the alternate endpoint of the stomach body.

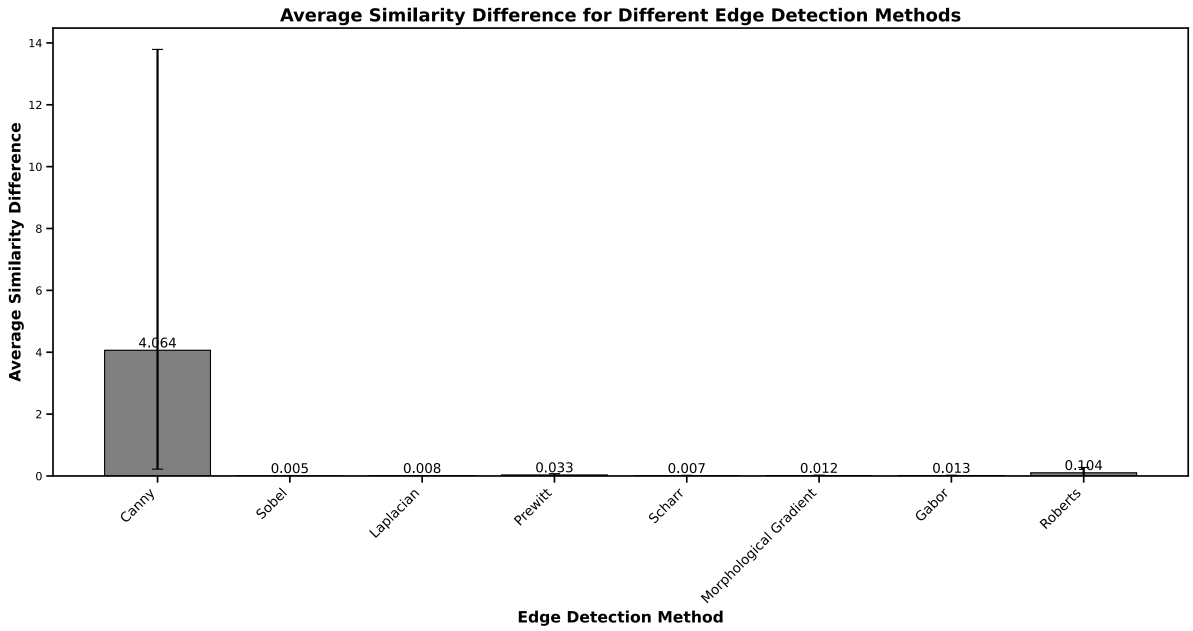

Figure S20. Similarity differences of each edge detection algorithm.

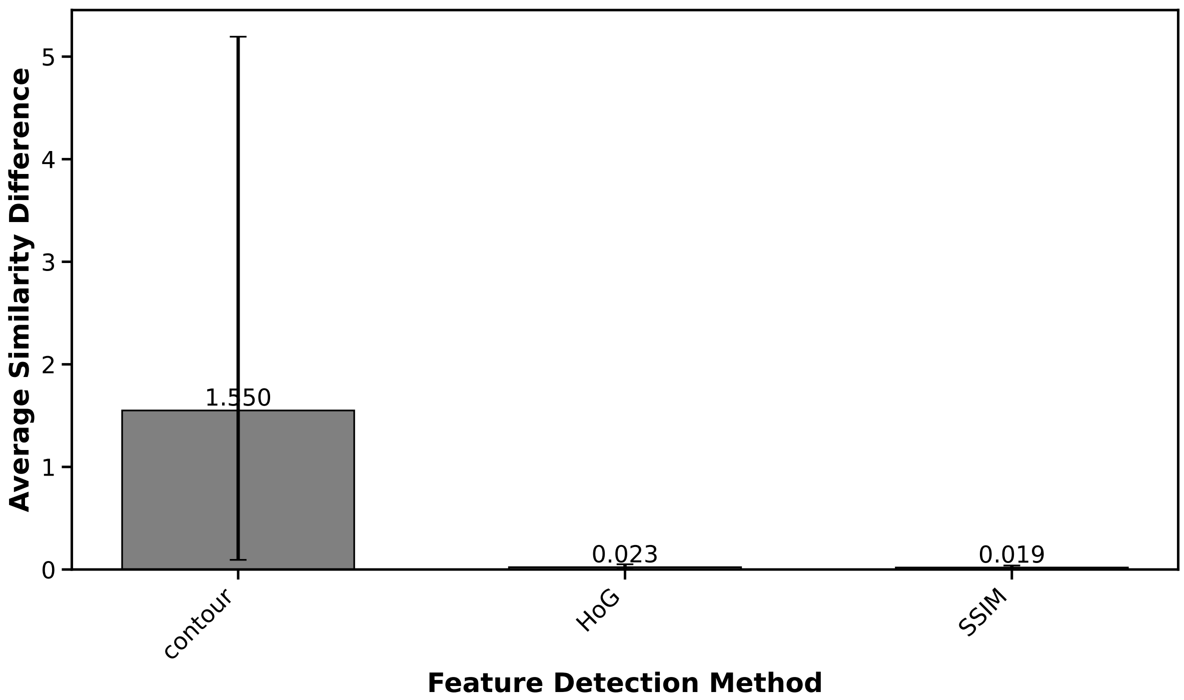

Figure S21. Similarity differences of each feature match algorithm.

Note S11. DispNet

Once the navigation endpoint is detected, its precise spatial coordinates must be determined. While the x and y coordinates can be estimated directly from the capsule's location in the image plane, obtaining the z coordinate requires reliable depth estimation. In this study, we adopted the DispNet architecture for this purpose.

DispNet is designed with multiple ResNet blocks, which increase network depth and enhance the ability to learn and represent complex spatial features. As shown in Figure S22a, the model follows an encoder–decoder paradigm composed of convolutional layers, ResNet blocks, and non-linear activations.

The encoder begins with a 3×3 convolutional layer with 64 filters (C64) consisting of convolution + batch normalization (BN) + rectified linear unit (ReLU) activation, followed by a max-pooling (MP) layer to reduce spatial resolution. This is followed by a sequence of ResNet blocks—RB64, RB128, RB256, and RB512—each containing two convolutional layers with BN and ReLU activation, and a skip connection that preserves low-level spatial information.

The decoder progressively upsamples features while reducing the number of filters through convolutional layers with exponential linear unit (ELU) activation: C256, C128, C64, and C32. A final convolutional layer C16 with 16 filters and sigmoid activation produces the normalized disparity map. Skip connections between encoder and decoder layers facilitate feature reuse and improve gradient flow.

For notation, RBk denotes a ResNet block with k filters, and Ck denotes a 3×3 convolutional layer with k filters. BN, ReLU, and ELU refer to batch normalization, rectified linear unit activation, and exponential linear unit activation, respectively.

A pre-trained DispNet model[21] was employed to infer depth from the capsule's viewpoint. The resulting depth map is shown in Figure S22c, where the red star marks the point with maximum depth. This value is used to compute the z coordinate of the detected endpoint, enabling accurate three-dimensional spatial localization.

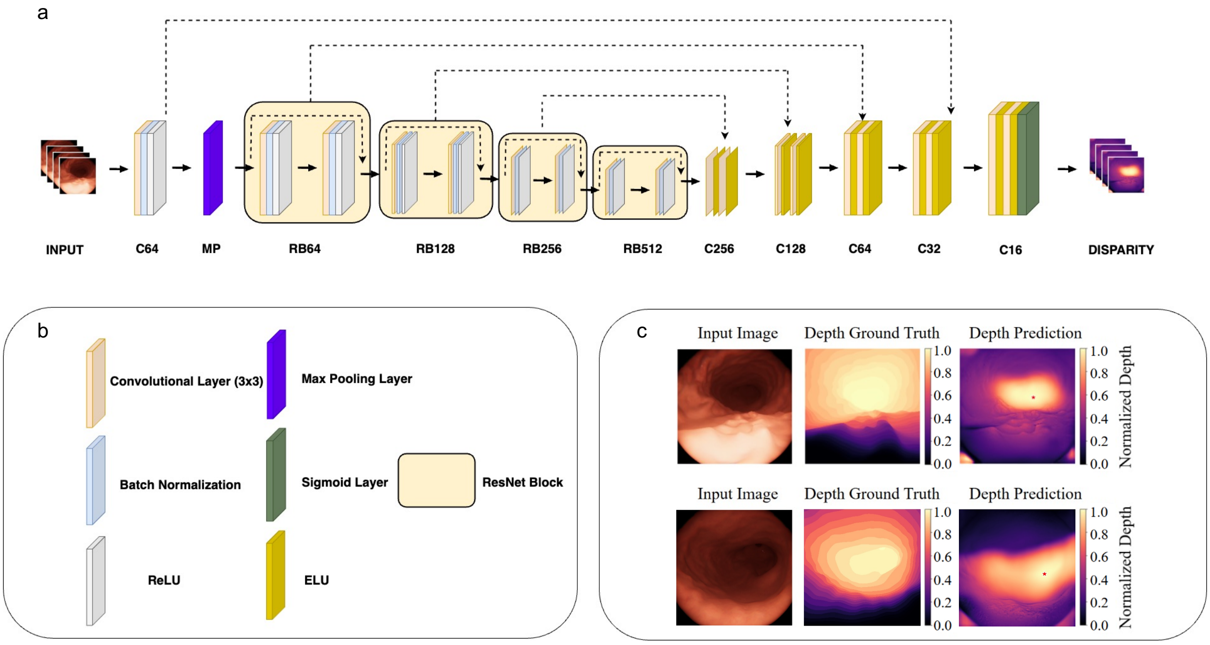

Figure S22. DispNet architecture and components.

Note S12. Reliability Analysis of the Perception Module under Challenging Scenarios

To rigorously evaluate the practical reliability of our perception system, we conducted experiments across three challenging scenarios: two cases involving varying off-axis tilts and one combining off-axis tilt with degraded illumination. The results shown in Figure S23 demonstrate that the edge-contour-depth fusion module—which integrates Canny edge detection and Hu moment invariants with the DispNet depth estimation architecture—successfully identifies anatomical landmarks and precisely estimates depth even when the endpoints are significantly offset from the optical center or subjected to poor lighting. This stable and accurate performance across all tested non-ideal conditions proves the high robustness of our edge-contour-depth fusion module against perspective distortions and illumination variations, confirming its reliability for maintaining autonomous navigation continuity in diverse clinical environments.

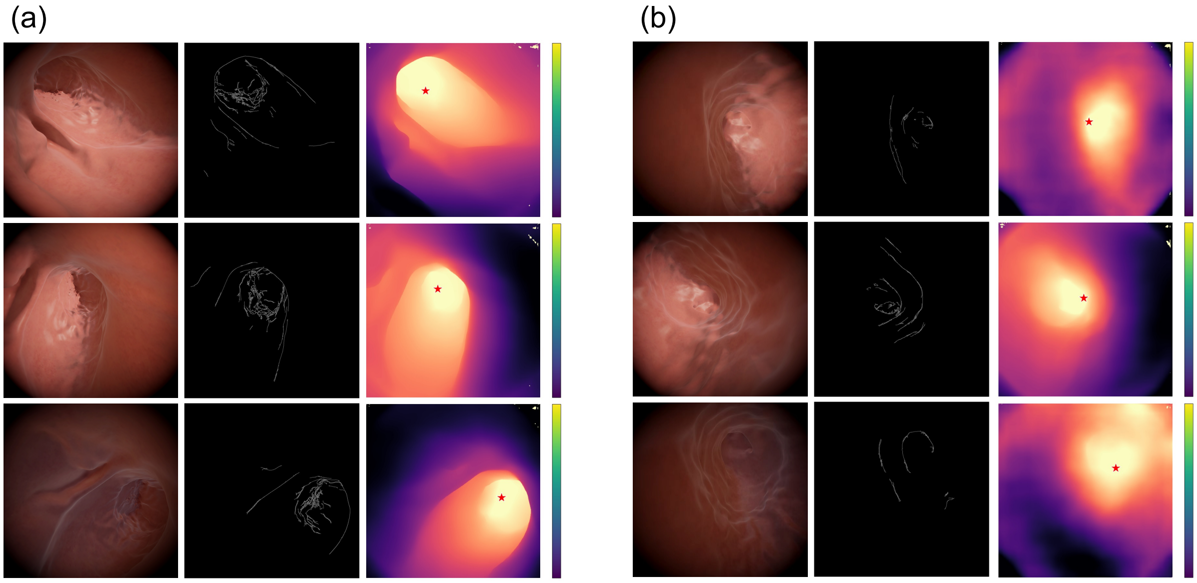

Figure S23. Performance of the landmark identification and depth estimation under off-axis tilts and low-illumination scenarios (a) results for End Point 1 (b) results for End Point 2.

Note S13. Real-Time Latency Compensation Mechanism

To bridge the sim-to-real gap, the ADP controller compensates for a ~30ms system latency by utilizing its learned transition dynamics for forward state prediction. As illustrated in Figure S24, although sensor data received at $t = 50$ ms is outdated (S_{20}) due to the 30 ms sensing delay, the ADP controller performs a forward prediction for the state at $t = 100$ ms (S_{100}). By optimizing the control command for this anticipated state, the action dispatched at $t = 70$ ms executes exactly at $t = 100$ ms (due to the 30 ms actuation lag). This proactive mechanism ensures that control inputs perfectly align with the capsule's actual physical environment upon execution, effectively neutralizing the destabilizing effects of latency to maintain robust navigation accuracy.

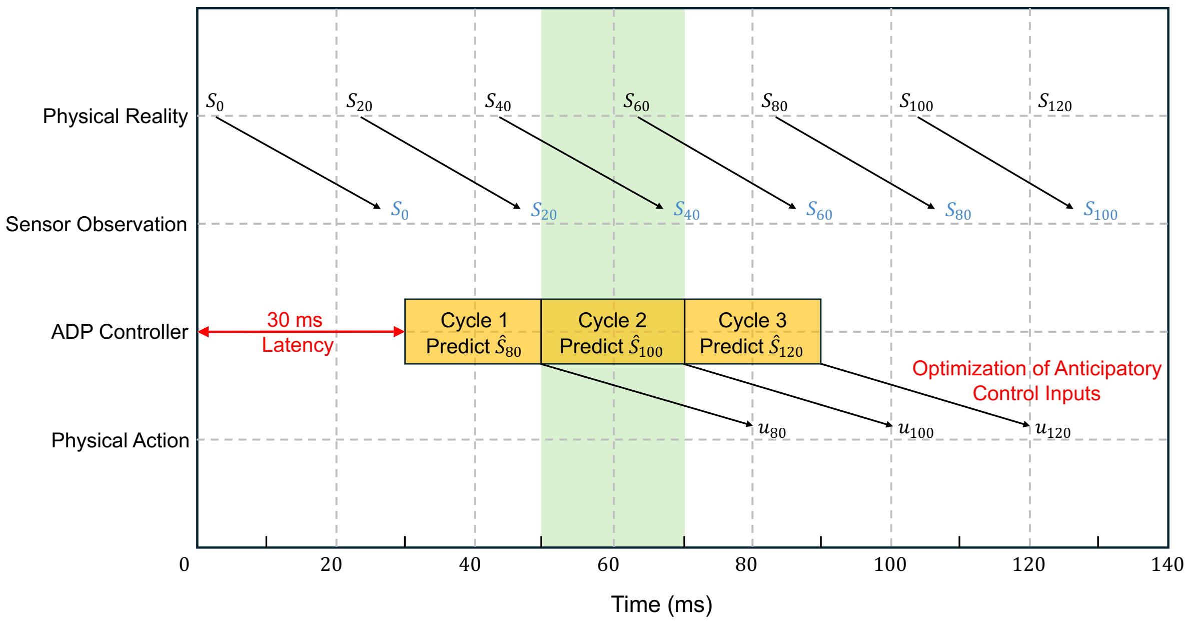

Figure S24. Timing diagram of the real-time latency compensation mechanism in the ADP controller.

Note S14. Deep Reinforcement Learning

Reinforcement learning (RL) is a machine-learning paradigm in which agents learn optimal behaviors through direct interaction with an environment, guided by reward signals rather than labelled datasets. By iteratively exploring state-action spaces and adjusting policies based on cumulative rewards, RL offers a principled framework for adaptive decision-making in dynamic, partially observable environments. It has achieved notable success in domains such as game playing, robotic control, and autonomous navigation. Despite this progress, classical RL algorithms often struggle to scale to high-dimensional state spaces and continuous action domains, owing to limited sample efficiency and instability during training.

DRL algorithm addresses these challenges by integrating deep neural networks with RL algorithms, enabling the extraction of rich features from high-dimensional sensory inputs and the approximation of complex value or policy functions. A seminal example is the DQN, which integrates convolutional neural networks with Q-learning to learn an action-value function $Q_\theta(s, a)$ directly from raw visual input[22]. In Q-learning, a value-based RL method, the optimal policy π^* is derived from the optimal action-value function $Q^*(s, a)$according to

$$a(s) = \arg\max_a Q_\theta\,(s, a) \tag{14}$$

with updates guided by the Bellman optimality equation. Q-learning is typically implemented in an off-policy setting, such that experience collected under arbitrary exploration policies can be reused for policy evaluation and improvement.

In contrast, SAC adopts an off-policy actor-critic formulation that maximizes a stochastic policy's expected return while regularizing it with an entropy term

$$H(P) = E_{x\sim P}[-\log P\,(x)] \tag{15}$$

where $P(x)$ is the policy's probability distribution over actions. The entropy bonus encourages exploration by penalizing over-confident action selection. The optimal entropy-regularized policy is obtained as

$$\pi^* = \arg\max_{\pi E_{\tau\sim\pi}} \left[\sum_{t=0}^{\infty} \gamma^t \left(R(s_t, a_t, s_{t+1}) + \alpha H\big(\pi(\cdot\,|s_t)\big)\right)\right] \tag{16}$$

where α controls the trade-off between reward maximisation and entropy.

PPO is a widely adopted on-policy algorithm valued for its implementation simplicity and robustness across diverse tasks. PPO constrains policy updates to prevent destructive deviations from the current policy π_{θ_k}, striking a balance between sufficient exploration and training stability. The update rule seeks

$$\theta_{k+1} = \arg\max_\theta E_{s,a\sim\pi_k}\,[L(s, a, \theta_k, \theta)] \tag{17}$$

where the clipped surrogate objective

$$L(s, a, \theta_k, \theta) = \min\left(\frac{\pi_\theta(a|s)}{\pi_{\theta_k}(a|s)} A^{\pi_{\theta_k}}(s, a),\ g\big(\epsilon, A^{\pi_{\theta_k}}(s, a)\big)\right) \tag{18}$$

uses the function

$$g(\epsilon, A) = \begin{cases} (1+\epsilon)A & A \geq 0 \\ (1-\epsilon)A & A < 0 \end{cases} \quad (19)$$

to clip the probability ratio, limiting the policy's divergence by a small hyperparameter ϵ. This approach enables multiple epochs of stochastic gradient descent on the same batch of simulated experience while maintaining trust-region-like stability.

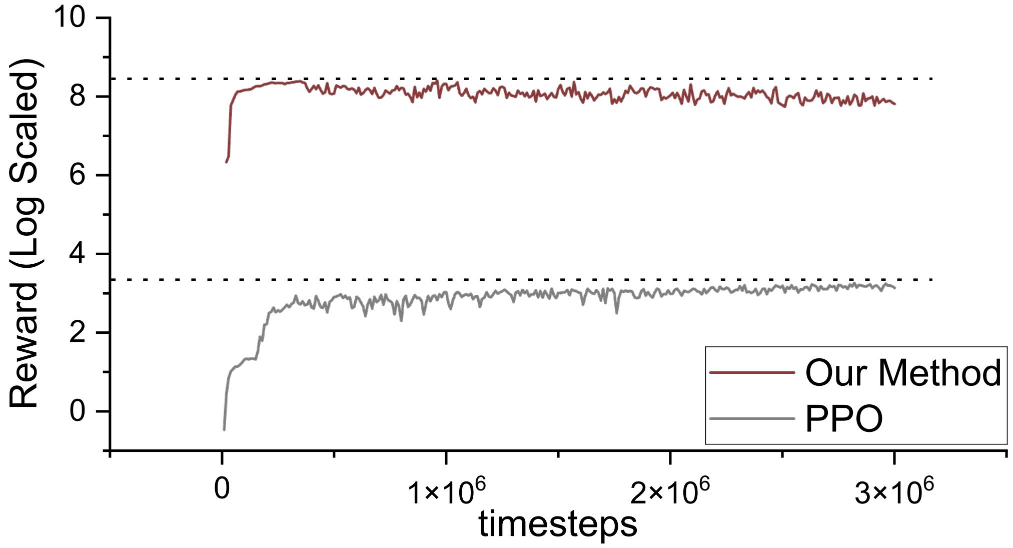

Figure S25. Comparison of reward convergence between vanilla PPO and the proposed AL-DRL framework within the training environment.

Table S2. Morphological characteristics and diversity of the eight patient-derived stomach models

Stomach	Shape	Capacity (mL)	GC / LC Length (cm)
stomach 1	J-shaped	680	18.9/13.0
stomach 2	Cylindrical	471	21.2/15.6
stomach 3	Crescentic	801	27.0/22.6
stomach 4	Reversed L	502	22.7/17.1
stomach 5	Cylindrical	471	25.6/19.3
stomach 6	J-shaped	569	22.2/17.6
stomach 7	J-shaped	610	21.7/14.6
stomach 8	Reversed L	557	26.8/20.1

Note: GC denotes the Greater Curvature of stomach; LC denoted the Lesser Curvature of stomach

Table S3. Comparison of edge detection and feature matching methods on stomach 1

Methods	Contour Difference	HOG Difference	SSIM Difference	Edge Detection Average
Canny	41.327	0.039	0.004	13.790
Sobel	0.000	0.001	0.003	0.001
Laplacian	0.000	0.000	0.001	0.001
Prewitt	0.217	0.004	0.003	0.075
Scharr	0.000	0.001	0.003	0.001
Morphological Gradient	0.000	0.001	0.001	0.001
Gabor	0.000	0.013	0.005	0.006
Roberts	0.005	0.005	0.002	0.004
Feature Match Average	5.194	0.008	0.003	

Table S4. Comparison of edge detection and feature matching methods on stomach 2

Methods	Contour Difference	HOG Difference	SSIM Difference	Edge Detection Average
Canny	0.700	0.011	0.008	0.239
Sobel	0.000	0.008	0.038	0.015
Laplacian	0.000	0.012	0.003	0.005
Prewitt	0.042	0.000	0.007	0.016
Scharr	0.000	0.006	0.031	0.012
Morphological Gradient	0.000	0.001	0.002	0.001
Gabor	0.000	0.023	0.009	0.011
Roberts	0.815	0.004	0.002	0.274
Feature Match Average	0.195	0.008	0.012	

Table S5. Comparison of edge detection and feature matching methods on stomach 3

Methods	Contour Difference	HOG Difference	SSIM Difference	Edge Detection Average
Canny	7.239	0.117	0.037	2.464
Sobel	0.000	0.006	0.009	0.005
Laplacian	0.000	0.010	0.022	0.011
Prewitt	0.006	0.012	0.034	0.017
Scharr	0.000	0.009	0.002	0.004
Morphological Gradient	0.000	0.003	0.022	0.008
Gabor	0.000	0.072	0.006	0.026
Roberts	0.786	0.002	0.022	0.270
Feature Match Average	1.004	0.029	0.019	

Table S6. Comparison of edge detection and feature matching methods on stomach 4

Methods	Contour Difference	HOG Difference	SSIM Difference	Edge Detection Average
Canny	3.758	0.012	0.013	1.261
Sobel	0.001	0.004	0.008	0.004
Laplacian	0.001	0.001	0.015	0.006
Prewitt	0.032	0.001	0.017	0.017
Scharr	0.002	0.004	0.011	0.005
Morphological Gradient	0.000	0.004	0.019	0.008
Gabor	0.000	0.008	0.000	0.003
Roberts	0.452	0.004	0.007	0.154
Feature Match Average	0.531	0.005	0.011	

Table S7. Comparison of edge detection and feature matching methods on stomach 5

Methods	Contour Difference	HOG Difference	SSIM Difference	Edge Detection Average
Canny	0.593	0.019	0.049	0.220
Sobel	0.001	0.001	0.002	0.001
Laplacian	0.001	0.000	0.029	0.010
Prewitt	0.099	0.006	0.045	0.050
Scharr	0.001	0.001	0.006	0.003
Morphological Gradient	0.001	0.005	0.035	0.014
Gabor	0.000	0.038	0.007	0.015
Roberts	0.056	0.010	0.021	0.029
Feature Match Average	0.094	0.010	0.024	

Table S8. Comparison of edge detection and feature matching methods on stomach 6

Methods	Contour Difference	HOG Difference	SSIM Difference	Edge Detection Average
Canny	34.121	0.122	0.025	11.422
Sobel	0.000	0.004	0.007	0.004
Laplacian	0.000	0.004	0.005	0.003
Prewitt	0.080	0.001	0.005	0.029
Scharr	0.000	0.003	0.012	0.005
Morphological Gradient	0.000	0.019	0.019	0.013
Gabor	0.011	0.013	0.005	0.010
Roberts	0.124	0.019	0.006	0.050
Feature Match Average	4.292	0.023	0.010	

Table S9. Comparison of edge detection and feature matching methods on stomach 7

Methods	Contour Difference	HOG Difference	SSIM Difference	Edge Detection Average
Canny	0.593	0.019	0.049	0.220
Sobel	0.001	0.001	0.002	0.001
Laplacian	0.001	0.000	0.029	0.010
Prewitt	0.099	0.006	0.045	0.050
Scharr	0.001	0.001	0.006	0.003
Morphological Gradient	0.001	0.005	0.035	0.014
Gabor	0.000	0.038	0.007	0.015
Roberts	0.056	0.010	0.021	0.029
Feature Match Average	0.094	0.010	0.024	

Table S10. Comparison of edge detection and feature matching methods on stomach 8

Methods	Contour Difference	HOG Difference	SSIM Difference	Edge Detection Average
Canny	7.608	0.211	0.129	2.650
Sobel	0.005	0.001	0.007	0.004
Laplacian	0.004	0.023	0.014	0.014
Prewitt	0.011	0.037	0.042	0.030
Scharr	0.005	0.021	0.038	0.021
Morphological Gradient	0.004	0.048	0.030	0.028
Gabor	0.002	0.043	0.032	0.026
Roberts	0.038	0.025	0.016	0.026
Feature Match Average	0.960	0.051	0.039	

Table S11. Edge detection algorithms summary

stomach model	Canny	Sobel	Laplacian	Prewitt	Scharr	Morphological Gradient	Gabor	Roberts
stomach 1	13.79	0.001	0.001	0.075	0.001	0.001	0.006	0.004
stomach 2	0.239	0.015	0.005	0.016	0.012	0.001	0.011	0.274
stomach 3	2.464	0.005	0.011	0.017	0.004	0.008	0.026	0.27
stomach 4	1.261	0.004	0.006	0.017	0.005	0.008	0.003	0.154
stomach 5	0.468	0.006	0.01	0.027	0.007	0.024	0.011	0.022
stomach 6	11.422	0.004	0.003	0.029	0.005	0.013	0.01	0.05
stomach 7	0.22	0.001	0.01	0.05	0.003	0.014	0.015	0.029
stomach 8	2.65	0.004	0.014	0.03	0.021	0.028	0.026	0.026

Table S12. Feature match algorithms summary

Stomach Model	Contour	HoG	SSIM
stomach 1	5.194	0.008	0.003
stomach 2	0.195	0.008	0.012
stomach 3	1.004	0.029	0.019
stomach 4	0.531	0.005	0.011
stomach 5	0.133	0.047	0.036
stomach 6	4.292	0.023	0.01
stomach 7	0.094	0.01	0.024
stomach 8	0.96	0.051	0.039

References

1 Lv, C., Lin, W. & Zhao, B. Voxel structure-based mesh reconstruction from a 3D point cloud. *IEEE Transactions on Multimedia* **24**, 1815-1829 (2021).

2 Charreyron, S. L. *et al.* Modeling electromagnetic navigation systems. *IEEE Transactions on Robotics* **37**, 1009-1021 (2021).

3 Petruska, A. J., Edelmann, J. & Nelson, B. J. Model-based calibration for magnetic manipulation. *IEEE Transactions on Magnetics* **53**, 1-6 (2017).

4 Mahoney, A. W. & Abbott, J. J. Five-degree-of-freedom manipulation of an untethered magnetic device in fluid using a single permanent magnet with application in stomach capsule endoscopy. *The International Journal of Robotics Research* **35**, 129-147 (2016).

5 Xu, Y., Li, K., Zhao, Z. & Meng, M. Q.-H. A novel system for closed-loop simultaneous magnetic actuation and localization of WCE based on external sensors and rotating actuation. *IEEE Transactions on Automation Science and Engineering* **18**, 1640-1652 (2020).

6 Chen, W., Sui, J. & Wang, C. Magnetically actuated capsule robots: A review. *IEEE Access* **10**, 88398-88420 (2022).

7 Ciuti, G., Valdastri, P., Menciassi, A. & Dario, P. Robotic magnetic steering and locomotion of capsule endoscope for diagnostic and surgical endoluminal procedures. *Robotica* **28**, 199-207 (2010).

8 Norton, J. C. *et al.* Intelligent magnetic manipulation for gastrointestinal ultrasound. *Science robotics* **4**, eaav7725 (2019).

9 Balakrishnan, S., Ding, J. & Lewis, F. L. Issues on stability of ADP feedback controllers for dynamical systems. *IEEE Transactions on Systems, Man, and Cybernetics, Part B (Cybernetics)* **38**, 913-917 (2008).

10 Shouran, M. & Elgamli, E. Design and implementation of Butterworth filter. *Int. J. Innov. Res. Sci. Eng. Technol* **9**, 7975-7983 (2020).

11 Su, S. *et al.* AMagPoseNet: Real-Time Six-DoF Magnet Pose Estimation by Dual-Domain Few-Shot Learning From Prior Model. *IEEE Transactions on Industrial Informatics* **19**, 9722-9732 (2023). https://doi.org/10.1109/TII.2022.3233675

12 Pham, L. N., Steiner, J. A., Leang, K. K. & Abbott, J. J. Soft endoluminal robots propelled by rotating magnetic dipole fields. *IEEE Transactions on Medical Robotics and Bionics* **2**, 598-607 (2020).

13 Ali, A. J. & Abbas, A. F. Applications of Numerical Integrations on the Trapezoidal and Simpson's Methods to Analytical and MATLAB Solutions. *Mathematical Modelling of Engineering Problems* **9** (2022).

14 Pham, D. M. & Aziz, S. M. in *2014 IEEE Ninth International Conference on Intelligent Sensors, Sensor Networks and Information Processing (ISSNIP).* 1-6 (IEEE).

15 Wu, X. *et al.* Wearable magnetic locating and tracking system for MEMS medical capsule. *Sensors and Actuators A: Physical* **141**, 432-439 (2008).

16 Kucuk, S. & Bingul, Z. *Robot kinematics: Forward and inverse kinematics*. Vol. 1 (INTECH Open Access Publisher London, UK, 2006).

17 Canny, J. A computational approach to edge detection. *IEEE Transactions on pattern analysis and machine intelligence*, 679-698 (1986).

18 Hu, M.-K. Visual pattern recognition by moment invariants. *IRE transactions on information theory* **8**, 179-187 (1962).

19 Lowe, D. G. Distinctive image features from scale-invariant keypoints. *International journal of computer vision* **60**, 91-110 (2004).

20 Wang, Z., Bovik, A. C., Sheikh, H. R. & Simoncelli, E. P. Image quality assessment: from error visibility to structural similarity. *IEEE transactions on image processing* **13**, 600-612 (2004).

21 Ozyoruk, K. B. *et al.* EndoSLAM dataset and an unsupervised monocular visual odometry and depth estimation approach for endoscopic videos. *Medical image analysis* **71**, 102058 (2021).

22 Mnih, V. *et al.* Human-level control through deep reinforcement learning. *nature* **518**, 529-533 (2015).
